# Analysis of genome-wide association study data using the protein knowledge base

**DOI:** 10.1186/1471-2156-12-98

**Published:** 2011-11-13

**Authors:** Sara Ballouz, Jason Y Liu, Martin Oti, Bruno Gaeta, Diane Fatkin, Melanie Bahlo, Merridee A Wouters

**Affiliations:** 1 Structural and Computational Biology Division, Victor Chang Cardiac Research Institute, Darlinghurst, NSW, 2010, Australia; 2 School of Computer Science and Engineering, University of New South Wales, Kensington, NSW, 2052, Australia; 3 Centre for Molecular and Biomolecular Informatics, Radboud University Nijmegen Medical Centre, Nijmegen, The Netherlands; 4 School of Medical Sciences, University of New South Wales, Kensington, NSW, 2052, Australia; 5 Molecular Cardiology and Biophysics Division, Victor Chang Cardiac Research Institute, Darlinghurst, NSW, 2010, Australia; 6 Bioinformatics Division, The Walter and Eliza Hall Institute of Medical Research, Parkville, VIC, 3052, Australia; 7 School of Life and Environmental Sciences, Deakin University, Geelong, VIC, 3217, Australia

## Abstract

**Background:**

Genome-wide association studies (GWAS) aim to identify causal variants and genes for complex disease by independently testing a large number of SNP markers for disease association. Although genes have been implicated in these studies, few utilise the multiple-hit model of complex disease to identify causal candidates. A major benefit of multi-locus comparison is that it compensates for some shortcomings of current statistical analyses that test the frequency of each SNP in isolation for the phenotype population versus control.

**Results:**

Here we developed and benchmarked several protocols for GWAS data analysis using different *in-silico *gene prediction and prioritisation methodologies. We adopted a high sensitivity approach to the data, using less conservative statistical SNP associations. Multiple gene search spaces, either of fixed-widths or proximity-based, were generated around each SNP marker. We used the candidate disease gene prediction system *Gentrepid *to identify candidates based on shared biomolecular pathways or domain-based protein homology. Predictions were made either with phenotype-specific known disease genes as input; or without *a priori *knowledge, by exhaustive comparison of genes in distinct loci. Because *Gentrepid *uses biomolecular data to find interactions and common features between genes in distinct loci of the search spaces, it takes advantage of the multi-locus aspect of the data.

**Conclusions:**

Results suggest testing multiple SNP-to-gene search spaces compensates for differences in phenotypes, populations and SNP platforms. Surprisingly, domain-based homology information was more informative when benchmarked against gene candidates reported by GWA studies compared to previously determined disease genes, possibly suggesting a larger contribution of gene homologs to complex diseases than Mendelian diseases.

## Background

The identification of genes implicated in human disease enables an understanding of disease mechanisms and is essential for the development of diagnostics and therapeutics. Many associations have now been identified from GWA studies. As of September 2011, the HuGE database http://www.hugenavigator.net contained 6164 associations from 1019 published GWAS. These methods have led to the discovery of several novel genes for complex diseases. However GWAS have not proved as powerful as originally hoped with approximately 3061 genes reported or identified, suggesting more may be gleaned by careful reanalysis of the data.

GWAS are designed to identify common genetic risk factors of complex diseases and quantitative traits, that are believed to be the result of multiple genetic and environmental factors [1]. GWAS use high-throughput genotyping platforms, such as SNP chips, which carry hundreds of thousands of SNP markers. Even with multiple marker testing, GWAS have greater statistical power to detect genetic variants that increase disease risks than linkage analysis [2], but hundreds of SNPs may be identified. In order to make sense of the large amount of data acquired, most published GWAS list only the top 20 to 50 most significant SNPs and their nearest gene using the "most significant SNPs/genes" approach [3], while ignoring the remaining SNPs [4-6]. A highly stringent significance threshold attempts to correct for the number of false positives, but this conservative statistical approach combined with the selection of the nearest-neighbouring gene to the significant SNP still has several limitations.

Phenotypes influenced by multiple genetic and environmental factors, or those with uncommon and small effect variants, are not detected after adjustment for multiple testing [7], thereby introducing a potentially high false negative rate to the study. Also, variants with larger effects might not always rank among the top markers reported when taking the most-significant SNP approach [7,8].

A further conundrum for GWAS has been the lack of genetic signals recovered to explain the genetic heritability of many diseases, implying that much has been missed due to the limitations of GWAS methodology [9]. This missing heritability may be due to the heterogeneous population studied, for example the disease may be caused by multiple rare variants; the fact that SNPs are tested in isolation, for example if important gene-gene interactions occur; inability to control the environment of the patient population, for example gene-environmental interactions may be important; or gaps in SNP chip coverage for some regions of the genome.

Furthermore, tagged SNPs on the platforms used in the studies are potentially only in linkage disequilibrium (LD) with the causal SNPs and further replication studies and sequencing is required to identify the actual causal variant. With the advent of next-generation sequencing, rapid follow up of multiple candidate markers or genes is possible.

The methods typically used by researchers to select genes associated with the significant SNP assume the disease-associated SNP is either resident in, or adjacent to, the disease gene. But the genetic architecture of the genome is still not well understood: work on long range gene regulation [10] suggests distal *cis*-acting elements can control genes that are not directly adjacent to the regulatory region of the gene. For instance fibroblast growth factor 8, *FGF8*, is controlled by regulatory elements within and beyond the neighbouring gene *FBXW4 *[11]. Therefore, the disease gene may be near the significant or causal SNP but may *not *be the closest gene to it, i.e. the causal SNP is in a regulatory region that acts distally on the disease-causing gene. In these cases, the simplistic approach currently used for SNP-to-gene mapping is limiting and the search space should be extended to include additional nearby genes that may play a role in the phenotype.

Clearly new approaches are required to utilize the valuable but noisy data from GWAS. In order to avoid "throwing the baby out with the bathwater", the statistical significance threshold can be decreased to study a much larger sample of SNPs which may potentially be associated with the disease. Although, this reduces the power of the study, these less significant SNPs can then be sifted using other information. Several approaches have been suggested. Genetic information can be used to "weight" SNPs according to their plausibility. Information can be in the form of genome-wide linkage from population data [12,13]; or prior probabilities of association in significance calculations [14,15]. In addition to genetic data, biomolecular data such as information on protein function and protein-protein interactions can provide valuable information to distinguish associated loci from noise.

*Gentrepid *is a second generation candidate gene prediction system tool that draws on two types of functional data to group genes [16,17]. The Common Pathway Scanning (CPS) module is a Systems Biology method based on the assumption that common phenotypes are likely to be associated with proteins that partake in the same complex or pathway [18]. In other words, disease-causing genes for a specific phenotype are more likely to interact with other phenotype-specific disease genes [19,20]. Potential disease genes are predicted by identifying all proteins within phenotype-associated loci that are part of a pathway or complex.

The second *Gentrepid *module is Common Module Profiling (CMP), a technique based on the principle that candidate genes have similar functions to disease genes already determined for the phenotype [21]. A unique feature of CMP is that it uses domain-based comparative sequence analysis to identify proteins with potential functional similarity. In the field of candidate disease gene prediction, genes are often treated as single functional units, but translated proteins fold into discrete globular structures of limited size called domains [22]. Sequence-determined autonomous folding of domains into conserved compact three-dimensional structures is proposed to occur through hydrophobic collapse. A domain-based sequence comparison approach has several advantages over protein-based ones. Multiple domains, each with their own biochemical function, are often combined into a single gene to encode its entire function in a modular fashion similar to LEGO blocks [23-25]. Dissection of a gene into domains thus potentially provides a more fine-grained approach to functional assignment than can be achieved on a gene-by-gene basis. In addition, a particular isoform of a gene may be implicated. At the phenotypic level, functional clustering can be used to advantage as there are fewer building blocks than genes. The number of human genes stands at 25,000 to 35,000 [26], and the estimated size of the proteome ranges from 90,000 [27] to 1,000,000 [28]. In comparison, the fold repertoire of domains in the planetary proteome has been estimated at between 1000 and 5000 folds [29-31]. A domain-based approach also enables better detection and annotation of protein features. Because structure is conserved over sequence, domain-based sequence comparison searches have been shown to be more accurate than full-sequence searches [32]. Using the Pfam library of Hidden Markov models [29], domains can be assigned to approximately 69% of human proteins which allows functional inference for around 54% of the human genome. Prioritisation of the genes predicted from the modules is based on the statistical significance of the results.

Here we developed and benchmarked several protocols for analysing GWAS data effectively using the well-studied WTCCC data set on seven diseases. This data is employed in two manners: firstly using known disease genes for a particular phenotype to seed the search; and secondly using an agnostic approach which searches for *de novo *relationships between multiple loci. Predictions are then benchmarked against known disease genes, and genes suggested by the WTCCC study. The results show that analysis of more SNPs and consideration of more genes around each SNP replicate data from previous studies more effectively. The system was capable of extracting significantly associated genes from those of lower significance, as well as known and novel candidate disease genes using either *a priori *genetic knowledge or *de novo *analysis.

## Results

To test the ability of *Gentrepid *to select and prioritise valid disease gene candidates from the SNPs of GWAS, we performed a series of analyses on data from case-control studies from the WTCCC [33]. Most early GWAS used the Affymetrix chip set with approximately 500,000 known SNPs (Affy500k). We extracted 459,231 autosomal SNPs from the chip set for further analysis as detailed in the methods.

### Average number of SNP associations per phenotype

First we selected appropriate significance thresholds for GWA SNPs that are associated with the phenotypes of interest by increasing the cut-off of the Cochran-Armitage association *p*-values. Although the data quality varies depending on the phenotype, four consistent thresholds were used for ease of comparison: a weakly significant set (WS, *p_GWA _*≤ 10^-3^), a moderately-weak significant set (MWS, *p_GWA _*≤ 10^-4^), a moderately-high significant set (MHS, *P_GWA _*≤ 10^-5^), and a highly significant SNP set (HS, *p_GWA _*< 5 × 10^-7^). Table 1 summarizes the average number of SNPs above each of the significance thresholds that were associated with the phenotypes. On average, 30 highly significant SNPs were associated with a phenotype and this rose to over 800 SNPs for the weakly significant data. We then clustered co-located SNPs into what we termed an "associated locus" (See Methods). Significant SNPs show strong clustering, with 50-60% of significant SNPs clustering in phenotype-specific loci, with an average of 3 SNPs per cluster. The HS threshold had, on average, 7 associated loci per phenotype whereas the average number of associated loci for the WS threshold was over 400 (Table 1).

**Table 1:** Average number of SNPs, loci and genes per phenotypes used in this study with significant association *p *values and associated annotated genes in *Gentrepid*

			Level			
			
			WS	MWS	MHS	HS
			
			*p *≤ 1e-3	*p *≤ 1e-4	*p *≤ 1e-5	*p *< 5e-7
**SNPs**			804.29	160.29	56.71	29.14

**Loci**			446.86	84.43	18.71	7.29

**Total Genes**	*BY*	1 Mbp	3875.57	870.86	175.29	87.43
		0.5 Mbp	2140.00	477.29	106.00	57.29
		0.1 Mbp	654.57	148.43	43.71	23.00
	*NN*	Adjacent	1412.14	292.43	62.29	26.14
		Nearest	452.86	91.00	22.29	10.14
		Resident	198.71	42.57	11.43	5.43

**Annotated Genes**	*BY*	1 Mbp	2285.29	528.86	116.43	61.57
		0.5 Mbp	1275.57	300.43	73.14	41.57
		0.1 Mbp	426.43	103.43	32.00	16.57
	*NN*	Adjacent	803.14	172.00	40.71	17.57
		Nearest	285.71	59.00	15.57	6.14
		Resident	155.29	33.43	8.86	3.57

**Column abbreviations: **HS, highly significant; MHS, moderately-high significance; MWS, moderately-weak significance; WS, weakly significant. **Row abbreviations: **SNPs, average number of implicated SNPs per phenotype; Loci, average number of SNP clusters per phenotype; "Total Genes", the average number of genes per phenotype in the designated constructed pseudo-intervals; "Annotated Genes", the average number of genes per phenotype in the designated constructed pseudo-intervals with *Gentrepid *annotations.

### Gene selection around associated loci

**Figure 1:**
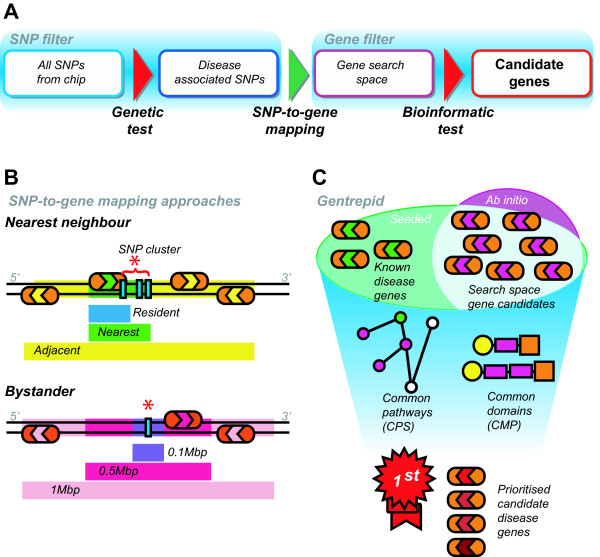
**Summary of GWAS analysis methodology**. (A) Double filter pipeline for GWAS data. The Genetic test filters for the disease-associated SNPs which are then mapped to genes. The Bioinformatics test, using *Gentrepid*, filters the genes for likely disease candidates. (B) SNP-to-gene approaches. The nearest neighbour approach consists of three sets: the *resident *set containing genes with SNPs internal to gene boundaries; the *nearest *set containing genes with SNPs internal or directly adjacent to the gene; and the *adjacent *set containing the four nearest genes to a SNP. The bystander approach consists of three sets where genes on both strands around SNPs were pooled from flanking intervals of fixed width. The sets include a 0.1 Mbp interval, 0.5 Mbp and 1 Mbp. Genes are represented as rounded rectangles and SNPs are marked as blue bars. (C) *Gentrepid *prediction method summary. A gene search space derived from GWAS data can be supplemented with known disease genes (*seeded*) or used stand-alone (*ab initio*). Genes involved in common pathways (CPS) or sharing common domains (CMP) within these search spaces are extracted by the system. Genes are prioritized based on the likelihood of genes with these properties occurring randomly.

To further investigate the relationship between phenotype-associated loci and nearby genes, genes were selected within a series of pseudo-intervals constructed around loci using one of two major assumptions (Figure 1). The first assumption, which gathers genes based on proximity to the associated locus, we termed the Nearest Neighbour (NN) approach. To enable discovery of genes subject to longer range regulation, we adopted an additional distance-based Bystander (BY) approach whereby genes were captured from an interval of fixed size created around each locus. For the NN approach, three sets of genes were created: a set containing genes with loci internal to the gene termed the *resident *set; a second set with loci directly adjacent to the gene, termed the *nearest *set; and a third set with the loci either resident in, or directly adjacent to, the four nearest genes, termed the *adjacent *set (Figure 1). NN sets are not distance based. For the BY approach, three pseudo-intervals of different sizes were tested: genes were pooled from flanking intervals of 0.1 Mbp, 0.5 Mbp or 1 Mbp in width around loci (Figure 1).

The 24 implicated search spaces per phenotype constructed using multiple SNP significance thresholds and gene selection methods ranged in size from 2 to 4431 genes: up to 10% of the genome. We have previously shown that candidate gene prediction by *Gentrepid *in such large search spaces is computationally feasible [17]. As shown in Table 1, more genes are associated with the phenotype-specific loci in the two larger bystander intervals (0.5 Mbp and 1 Mbp). However, the *adjacent *NN gene set usually contains more genes than the smallest BY interval for each phenotype (0.1 Mbp), as often one of the adjacent genes is located farther than the 50 Kbp distance threshold used (0.1 Mbp/2). Genes in the *adjacent *set are on average 362 Kbp (178-388 Kbp) away from the associated SNP, whilst genes in the *nearest *set are on average 90 Kbp (20-96 Kbp) away.

### Constraints on genomic coverage

**Figure 2:**
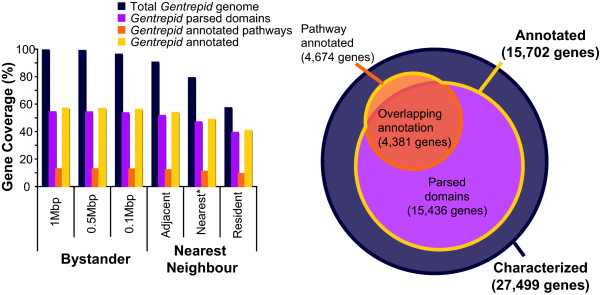
**Genomic coverage of the entire Affymetrix 500 K chip set SNPs (purple, left-most bar) and annotated genomic coverage of *Gentrepid *(other bars) when pseudo-intervals are constructed around the SNPs using each approach tested**. The bar graph on the left shows how many characterized genes in the genome are covered or represented for each approach tested. The approach most commonly used in GWAS, *nearest*, is indicated with an *. The Affy Chip set only covers 80% of the genes in the genome using this method. The BY approach has the higher total genome coverage, and this also holds when looking at the annotated coverage. The Venn diagram on the right shows the total overlap between the annotated (15,702) and characterized genes (27,499) in *Gentrepid*, with the greater portion of annotation from domain information. Parsed domains refer to the genes with Pfam domain information.

As a baseline, we wished to establish how genomic coverage by the Affy500K SNP chip set used in the WTCCC study depended on the approaches and assumptions used, and if these genes were represented in *Gentrepid *by associated pathways and domains. Figure 2 shows coverage of the human genome by the Affy500K chip set using the three gene selection methods for each of the NN and BY approaches tested. Here we define genes that are present in RefSeq [26] as "characterized" genes and those that have either a domain predicted through Pfam [29], or pathways and interactions partners in *Gentrepid *as "annotated". By selecting only the nearest gene to the associated SNP, as currently done in GWAS (*nearest *NN set), only 76% of characterized genes are associated with a SNP. Gene coverage increases to 90% if we associate nearest genes in the 3' and 5' direction on both strands with the SNP (*adjacent *NN set). Using the BY approach, gene coverage increases with interval size, ranging from 96% (0.1 Mbp) to 99.4% (1 Mbp) of characterized genes. SNPs on the Affy500K chip sets are randomly distributed across the genome, and ignore LD patterns. We confirmed this by observing that the density of SNPs on the chip sets is similar to the density of SNPs across the genome from dbSNP (data not shown). The Affy500K chip set's coverage of the genome has been shown to be poorer than other platforms that attempt to capture as much genetic variation as possible through LD-based tagging [34].

### Comparative overview of candidate gene predictions

To assess the ability of the two *Gentrepid *modules (CPS and CMP) to independently extract positional candidates from less significant data, we analysed the GWA-implicated pseudo-intervals chosen using both the NN and BY assumptions at the different levels of stringency. Two modes of input, referred to as "*seeded *mode" and "*ab initio *mode" were used to determine the common properties of phenotype-specific genes within the six gene sets for each disease. *Seeded *mode is assisted by phenotype-associated genes from OMIM [35] (Additional file 1, **Table S1**). The "known" disease genes were defined as those determined prior to GWAS of these diseases, and therefore are restricted to OMIM entries. *Ab initio *mode uses only genes pooled from the SNP-associated intervals: no additional genetic data beyond the GWA-implicated loci is required.

We compared the average number of significant predictions made by each of the modes and modules and the complementarity of these predictions (Table 2) across all search spaces tested. A CPS prediction was considered significant at *p *< 0.05, whilst CMP predictions were determined through scores/statistical tests described in detail in the methods section. *Ab initio *mode had, on average, more predictions when compared to *seeded *mode for the same gene search space set, indicating that there was novel information in the genetic data that was not represented by the known disease genes: a success for the GWAS methodology. In some instances, there were no predictions made by the *seeded *mode, suggesting earlier studies were either on the wrong track, or their results are limited to the studied family. Interesting differences were noted between the two bioinformatic methods depending on the significance of the SNPs used. Fewer predictions were made in the *nearest *and *resident *approaches of the HS and MHS thresholds, suggesting that the use of more generous thresholds may be detecting weaker effects. CPS *seeded *made more predictions than CMP *seeded*. CPS *ab initio *made, on average, more predictions than CMP *ab initio *for the WS and MWS data. For the MHS and HS thresholds, CMP *ab initio *made more predictions than CPS *ab initio *except for the HS *nearest *set where CMP *ab initio *made no predictions. For the stringent thresholds, the number of predictions was similar for each module.

**Table 2:** Average number of gene predictions made by *Gentrepid *modes and modules

Mode	SNP/gene		CPS				CMP				CPS + CMP			
		
	Approach		WS	MWS	MHS	HS	WS	MWS	MHS	HS	WS	MWS	MHS	HS
*Seeded*	*BY*	1 Mbp	29.14	6.00	2.29	0.71	15.43	3.86	0.71	0.43	42.86	9.43	3.00	1.14
		
		0.5 Mbp	12.29	5.29	2.00	1.29	9.00	2.86	0.57	0.29	20.71	7.71	2.57	1.57
		
		0.1 Mbp	6.71	3.00	1.71	1.00	3.14	1.00	0.29	0.00	9.57	3.57	1.86	1.00
	
	*NN*	Adjacent	19.14	6.00	1.57	1.43	6.86	1.57	0.43	0.00	25.29	7.00	1.86	1.43
		
		Nearest	10.86	2.57	1.43	1.00	3.00	0.86	0.29	0.00	13.71	3.29	1.57	1.00
		
		Resident	4.29	1.43	1.00	0.57	1.29	0.43	0.14	0.00	5.57	1.86	1.14	0.57

*Ab initio*	*BY*	1 Mbp	105.00	27.14	6.86	5.86	57.57	14.71	12.57	10.14	157.14	40.71	17.43	14.29
		
		0.5 Mbp	41.29	17.43	5.86	2.86	30.71	12.71	8.57	10.43	70.00	29.57	14.43	11.86
		
		0.1 Mbp	28.57	6.71	2.00	0.57	12.14	10.00	7.86	3.57	38.14	16.71	9.86	4.14
	
	*NN*	Adjacent	59.57	13.86	2.14	0.71	26.86	8.71	5.29	2.00	81.14	21.29	6.71	2.71
		
		Nearest	28.71	5.14	1.00	0.57	10.86	2.00	1.71	0.00	37.43	6.86	2.71	0.57
		
		Resident	13.00	2.57	0.29	0.00	9.71	0.86	0.57	0.00	21.00	3.14	0.86	0.00

*Seeded and ab initio*	*BY*	1 Mbp	105.00	27.14	6.86	6.57	72.43	18.43	13.29	10.57	**170.14**	**44.00**	**18.14**	**15.43**
		
		0.5 Mbp	41.43	17.43	5.86	3.57	39.57	15.57	9.14	10.71	**78.43**	**32.00**	**15.00**	**12.86**
		
		0.1 Mbp	28.57	7.00	2.29	1.29	15.29	11.00	8.14	3.57	**40.57**	**17.57**	**10.29**	**4.86**
	
	*NN*	Adjacent	59.57	13.86	2.29	1.71	33.43	10.29	5.71	2.00	**86.57**	**22.29**	**7.14**	**3.71**
		
		Nearest	28.71	5.29	1.71	1.14	13.86	2.86	2.00	0.00	**39.86**	**7.57**	**3.57**	**1.14**
		
		Resident	13.00	2.71	1.00	0.57	10.71	1.29	0.71	0.00	**22.00**	**3.71**	**1.71**	**0.57**

Each cell represents the number of predictions averaged across the seven phenotypes made by the mode and module. **Column abbreviations: **HS, highly significant; MHS, moderately-high significance; MWS, moderately-weak significance; WS, weakly significant; CPS, common pathway scanning module; CMP, common module profiling. **Row abbreviations: ***Seeded*, mode using known disease gene information as seeds; *Ab initio*, blind approach mode; BY, bystander approach; NN, nearest neighbour approach. The total number of predictions made by *Gentrepid *for each significance threshold and SNP/gene approach are bolded. All results have been filtered on significance. For CPS, pathways reach a significance threshold of *p *< 0.05 based on Fishers exact test. For CMP *seeded*, a threshold of 0.4. For CMP *ab initio*, a threshold of *χ^2^_max_unique _*> 105 and *χ^2 min ^*> 7.88 for multidomain proteins and *χ^2 min ^*> 100 for single domain proteins.

**Figure 3:**
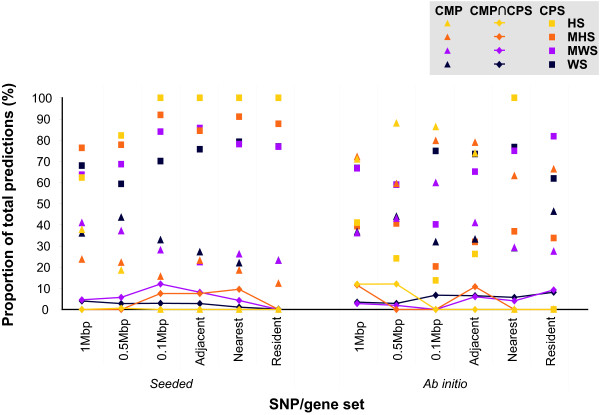
**Summary of *Gentrepid *predictions for the *seeded *and *ab initio *modes of the CPS and CMP modules averaged across phenotypes**. For each SNP-to-gene approach, the average proportion of the total predictions returned by CPS (represented as squares), CMP (represented as triangles), and the intersecting results from both modules (represented as diamonds with joining lines) are shown. The proportion of predictions refers to the ratio of the average number of genes predicted by each module to the average number of predictions made by both modules (i.e. CPS ∩ CMP). The four SNP sets employed are shaded yellow for HS, orange for MHS, purple for MWS and dark purple for WS. The proportion of predictions made by CPS in *seeded *mode was generally larger than the proportion predicted by CPS in *ab initio *mode. In *ab initio *mode, CMP makes the lion's share of predictions in search spaces with an intermediate number of annotated genes. For example, for the WS set which has the largest number of pseudo-intervals, the average size of the largest search space is 2285 annotated genes. CMP makes proportionally more predictions than CPS for the four smaller pseudo-intervals (total average gene set sizes of 155-803 annotated genes). For several of the HS sets no predictions were made and hence are superimposed on the x-axis. The average number of predictions per mode, module and WTCCC SNP set is shown in Table 2.

A summary of the proportion of the total number of significant predictions made by each module is represented in Figure 3. For *seeded *predictions, CPS made more predictions than CMP, as shown by the distinct data points on the left hand side of Figure 3. CPS predicted between 59-100% of total *seeded *predictions, while CMP only predicted between 12-43% of total *seeded *predictions. However, at most 12% of these predictions are common to the two modules (joined diamonds at bottom). In the *ab initio *analysis, CMP made a larger contribution to the number of predictions compared to the *seeded *mode. CPS predicted between 14-100% of *ab initio *predictions, while CMP predicted between 27-88% of *ab initio *predictions. But again, the two modules were relatively independent with 12% of these predictions in common. The percentage of *ab initio *predictions made by each module varied depending on the gene selection method and significance threshold, as shown by the less distinct separation of points on the right hand side of Figure 3. For the WS sets, CPS made more *ab initio *predictions; while for the MHS and HS sets, CMP made more predictions except for the smaller *nearest *and *resident *sets. The fewer predictions by CMP *ab initio *could reflect one of two things: true biological signals in complex diseases are missed in these smaller sets because candidate genes with long range effects are not included, or that the predictions made in the smaller sets with fewer genes fail to reach significance in our tests. *Seeded *and *ab initio *predictions are most congruent for CPS, with shared predictions comprising between 16-62% of total CPS predictions. For CMP, the predictions made by the *seeded *and *ab initio *modes are dissimilar, with 0-3% overlap in predictions. The congruency in predictions between CPS *seeded *and *ab initio *indicates that CPS works well as a search tool because the same pathways are being selected by the *ab initio *method without the *a priori *knowledge supplied to the *seeded *method. CPS is also a good discovery tool as there are novel predictions made by *ab initio *that were not detected by the *seeded *method. On the other hand, the fewer similar predictions made through the CMP *seeded *and *ab initio *modules might reflect differences in candidate gene significance filtering, or perhaps that CMP *ab initio *is making many more novel predictions and is a much stronger discovery tool for GWAS data. As the pathways utilized by CPS are often elucidated as part of the disease discovery process, the domain-based CMP approach may be a superior source of novel predictions for poorly characterised diseases when implemented using the agnostic *ab initio *approach.

### Benchmarking against validation sets

**Figure 4:**
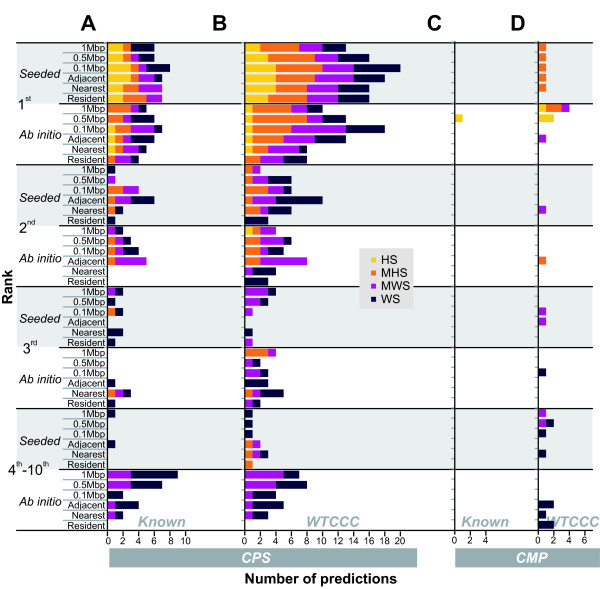
**Histogram of the ranks of genes predicted for validation sets across the SNP/gene search spaces: CPS predictions against the (A) Known disease gene set and (B) WTCCC-implicated gene candidates; CMP predictions against the (C) Known disease gene set and (D) WTCCC-implicated gene candidates**. The data sets are shaded based on the significance of the underlying SNP set: yellow for HS, orange for MHS, purple for MWS and dark purple for WS. In each set predictions made using known disease genes as seeds are shown on a grey background and *ab initio *predictions are shown on the white background. The graph shows that the priority assigned to a candidate gene prediction by *Gentrepid *agrees well with the significance of the underlying SNP. Predictions based on the most significant HS SNPs are clustered at the top of the figure (ranked first), showing prioritisation by CPS is effective. CMP, on the other hand, effectively ranks a handful of predictions made in *ab initio *mode when judged against WTCCC candidates. The majority of CMP predictions have not been previously detected. Ranks are displayed up until 10^th ^place.

**Figure 5:**
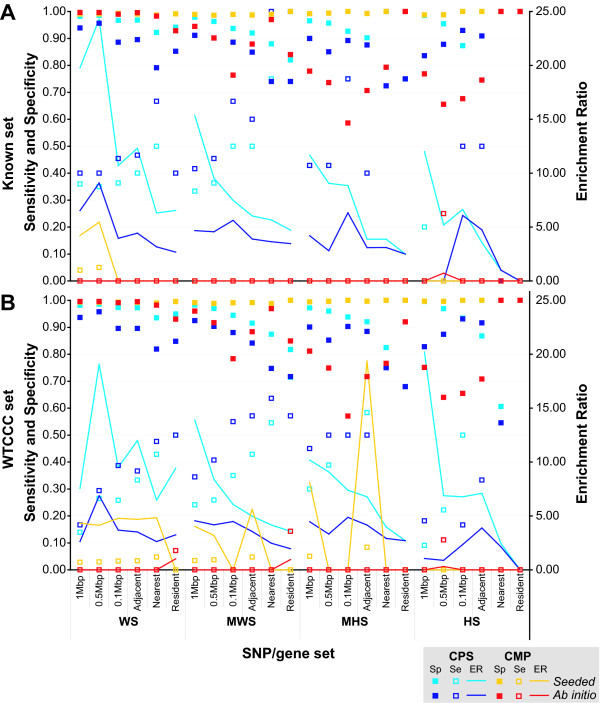
**Specificity, Sensitivity and Enrichment Ratios for validation sets across all phenotypes: (A) Known disease genes validation set and (B) WTCCC candidate genes validation set for alternate SNP/gene approaches across all thresholds**. Light blue depicts CPS *seeded*, dark blue-CPS *ab initio*, yellow-CMP *seeded *and red-CMP *ab initio*. On the primary axis, specificity is shown by shaded boxes and sensitivity by open boxes. On the secondary axis the enrichment ratios are shown as lines. For CPS, the sensitivity increased for the NN sets (*adjacent*, *nearest *and *resident*). For CMP, the sensitivity was low due to few predictions. For CPS and CMP, specificity decreased for the NN sets. The enrichment ratios decreased for CPS *seeded *for the NN sets, while for CPS *ab initio*, the highest enrichment ratio was for the 0.5 Mbp BY search space constructed from the WS SNP set, the 0.1 Mbp BY for the MWS and MHS sets, and the *adjacent *NN for the HS set. Some values are obscured, but have been listed in the supplementary Table S2 (Additional file 1).

As *Gentrepid *is intended as a discovery tool, there is currently no absolute way to determine if the candidate genes selected by the *Gentrepid *modules are indeed true positives without further genetic and molecular analyses on patients with the genotype. As a proxy, we tested *Gentrepid *on two datasets containing either known causal genes or genes that have a high probability of being causal. The first set consisted of known disease genes and loci from the phenotype data; whereas the second set of genes were implicated by the WTCCC as candidates. The ability of *Gentrepid *to extract and prioritise the genes in these sets was tested (Figure 4). In order to determine the overall performance of the modules being tested on the validation set, we calculated the sensitivity, specificity and enrichment ratios of each of the methods for each significance threshold (Figure 5).

Our first validation set consisted of 97 known disease genes collated from OMIM for the 7 diseases from the WTCCC study (Additional file 1, **Table S1**) Of these 97 genes, up to 29 were within WTCCC-implicated loci depending on the SNP-to-gene selection method employed and 7 of these genes were in highly significant loci (Additional file 1, **Table S2**). However the remaining known disease genes, constituting 70% of genes extracted from OMIM, were not in the search spaces at all. The lack of congruence between previous studies and the WTCCC data could be due to several factors which include differences in study design, differences in studied populations leading to allelic and locus heterogeneity, or true genetic differences. Our second validation set consisted of the WTCCC-implicated candidates, a total of 62 genes, from both significant and modestly associated SNPs [33] (Additional file 1, **Table S1**). Of these genes, 47 were within the search space, but only 39 were *Gentrepid *annotated genes. Of the 39 genes in at least one search space, 16 were in highly significant loci (Additional file 1, **Table S3**).

The ability of CPS to predict and prioritise known disease genes is shown in Figure 4A. A predicted gene is assigned an ordinal priority based on the statistical significance of the pathway it shares with other phenotype-implicated genes and thus has a rank equal to other candidates in the same pathway. The majority of known disease genes were in the highest ranked pathway for the phenotype. Known disease genes comprised 62% of all *seeded *predictions and 42% of all *ab initio *predictions. Most genes in the 0.1 Mbp and *adjacent *approaches were ranked 1^st^, but generally the gene selection method used had little effect on priority. Some deterioration of the signal is apparent for the least statistically significant data (WS), when the more demanding *ab initio *method is employed; or when larger search spaces are used. The ability of CPS to prioritise WTCCC phenotype-specific candidates is shown in Figure 4B. Despite being confronted with increasingly large search spaces, CPS is still able to extract biologically relevant genes from the increasingly less significant genetic data. Genes associated with the most statistically significant SNPs were primarily ranked first, constituting up to 66% of all predictions in *seeded *mode and 46% of all *ab initio *predictions. Of the 16 annotated WTCCC candidates in the HS sets: 4 candidates from the *adjacent *set were predicted by CPS *seeded *and given the top priority; 3 genes were predicted and given the top priority by CPS *ab initio*, and a fourth gene was ranked 2^nd^. Overall for CPS, genes in both validation sets were ranked first when the 0.1 Mbp or *adjacent *gene selection methods were used.

The ability of CMP to prioritise known disease genes is shown in Figure 4C. A predicted gene is assigned an ordinal priority based on its score in CMP *seeded*, and the χ^2 min ^score of CMP *ab initio*. Only 7 pairs of the phenotype-specific known disease genes share common domains, so CMP *seeded *was not expected to make many predictions based on the available input. Even so, only a single known gene was predicted by CMP *seeded*, *TCF2 *for the T2D phenotype which shares hepatocyte nuclear factor 1 domains HNF-1B_C (PF04812) and HNF-1_N (PF04814) with known disease gene *TCF1*. CMP *ab initio *predicted *CARD15*/*NOD2 *for the CD phenotype, but other predictions did not pass the required thresholds. The ability of CMP to prioritise WTCCC phenotype-specific candidates is shown in Figure 4D. CMP *seeded *only predicts *HHEX *for the T2D phenotype based on the homeobox domain it has in common with known disease genes *IPF1 *and *PAX4*. CMP *ab initio *predicted a total of 6 of the 39 WTCCC candidates, ranking the predicted genes 1^st ^to 10^th^. Overall, CMP prioritised the WTCCC validation set genes in the top ten in a manner that was in rough agreement with SNP significance.

Further to the prioritisation results, the specificity, sensitivity, and the enrichment ratio (ER) metrics allow for an overall quantitative comparison of the performance of the individual modes and modules (Figure 5, Additional file 1, **Table S4**). The sensitivity of the system ranged between 0.09 and 1. CPS in both *seeded *and *ab initio *mode had higher sensitivity scores compared to CMP. CPS *ab initio *generally had the highest sensitivity compared to the other modes and modules; holding true for both validation sets. Using the known WS set for validation, CPS *seeded *had a sensitivity that ranged between 0.35 to 0.50, while CPS *ab initio *was between 0.40 and 0.67. CPS had higher sensitivity in the NN gene selection sets compared to the BY sets. For CMP, the sensitivity was low due to few predictions. The specificity of the system ranged between 0.55 and 1. For CPS and CMP, specificity was less for the NN gene selection sets compared to the BY sets. For instance for the WS SNPs validated against known disease genes, CPS *seeded *had a specificity between 0.97 and 0.99 for the BY gene sets but only 0.92 to 0.97 for the NN gene sets. The ER of the system components varied between 1 (no enrichment) and 24.29 fold enrichment. For CPS *seeded *ERs decreased with NN gene set size, while for CPS *ab initio*, the maximal ER was for the 0.5 Mbp BY gene set for the WS SNPs, but the smaller 0.1 Mbp BY gene set for the MWS, MHS and HS SNP sets. Similar results were obtained with the WTCCC validation set. CMP *seeded *and *ab initio *benchmarked poorly against the known validation set, but CMP *seeded *performed much better on the WTCCC validation set. As for CPS, the largest ERs for CMP *seeded *were returned for the MWS and MHS data sets using the *adjacent *gene sets, and similar ERs for all the remaining approaches (except the *resident*) using the WS data set.

### Comparison to random controls

**Figure 6:**
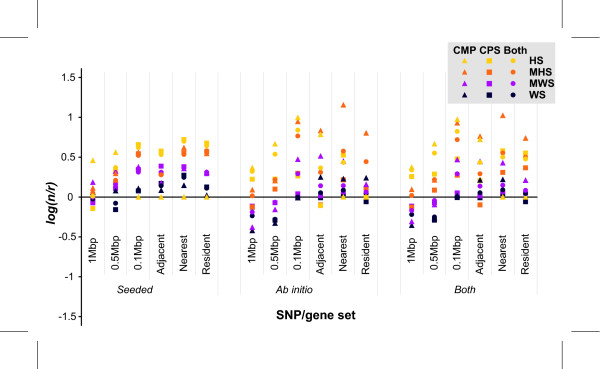
**Performance of the different modules on the GWAS data compared to randomly generated data**. Each point represents the log of the ratio of predictions for a phenotype-specific data set compared to a similar randomly generated SNP set. A positive ratio indicates better performance by the system. For the HS and MHS sets, the system performs better under most SNP/gene approaches, with the MHS set performing the best. In the MWS and WS sets, performance depends on the SNP/gene approach used to map the search spaces, with the NN gene set outperforming the BY sets. The *nearest*, *adjacent *and 0.1 Mbp approaches have the best performance.

As another test of the system, we compared predictions based on the WTCCC SNPs with predictions based on randomly generated SNP data. This test allows us to get a better handle on variables such as which gene selection sets are optimal, or if the signal-to-noise ratio begins to decrease as we decrease the significance level, as would be expected. We ran 1000 permutations of randomly selected SNPs for each disease, mode (*seeded/ab initio*) and module (CPS/CMP) set. As an indicator of performance, we calculated the log ratio of the number of predictions generated from the real data to the average from the randomly generated sets **(**Figure 6**)**. A positive ratio indicates better performance on real versus random data, a neutral score no difference from random, and a negative ratio poorer performance. For example, if the WTCCC SNPs are really phenotype-specific at a particular significance level, we would expect a larger number of predictions by the real data than by random SNPs.

When predictions made in *seeded *mode using phenotype-related SNPs were compared to predictions based on random SNPs across the different gene selection sets, the performance of the system was mode and module specific. CPS *seeded *performed best on the NN sets. CMP *seeded *performed best on BY sets, but CMP *ab initio *performed best on the NN sets. CPS *ab initio *was the worst performing module, mode combination. *Seeded *mode performs best across all SNP/gene approaches, whilst *ab initio *performs the best for the NN sets. CPS performed the best for the NN sets, while CMP performs well across all SNP/gene approaches.

When predictions made in *seeded *mode using phenotype-related SNPs were compared to predictions based on random SNPs across the significance thresholds, CPS had a positive ratio for the MWS-, MHS- and HS-implicated loci. CPS *ab initio *had a positive ratio for the MHS and HS sets. For CMP, both *seeded *and *ab initio *modes generally had positive ratios for the MHS and MWS set, neutral performance for the HS set, and a negative ratio for the WS set. In summary, the best performances were on the MHS and MWS thresholds across all the modules.

The poor performance of CPS on the WS sets in these tests using random data as compared to the benchmarks performed on previously discovered disease genes was surprising. Examining the random data shows that non-specific pathways generated a disproportionate number of CPS predictions which led to an increased rate of false positives. An example is the cytokine-cytokine receptor pathway from KEGG which retrieves generic cytokines which are not cognate ligands for the retrieved cytokine receptors. Using the MHS data, the system performs better than random when pathway data is available because of the higher statistical significance of the SNPs in this dataset, but the number of loci is diminishing to the point where it is not possible for CPS to make a prediction. This is also a reflection of the dependence of the analyses on the quality of current databases and annotations.

The same significance thresholds used in CMP *ab initio *across all the SNP/gene approaches do not take into account that the appropriate threshold may be search space dependent, as there are more genes in sets such as the 1 Mbp compared to the *adjacent*. Post-filtering the candidate genes based on the number of times they appeared in the random simulations was one approach we took to reduce the number of false positives in the phenotype-specific predictions. Fewer predictions were filtered from the NN sets for all phenotypes, which indicates there was most likely less noise in these search spaces. The gene predictions in the more selective sets were robust when filtered against the random simulations, suggesting genuine predictions. As in the case of CPS, CMP *ab initio *predictions require multiple loci which are fewer in the genetically more selective MHS and HS sets.

### Comparison to other systems

Our system focuses on the use of the protein knowledge base for predictions of candidate disease genes from implicated regions but there are other tools and methods that utilise alternate annotation information to perform predictions. Previously, we compared *Gentrepid *to 6 other candidate gene prediction systems using linkage analysis data against GWA results for type II diabetes [17]. Here, we compared the performance of our system on the GWAS data to two other candidate gene prediction tools currently available online: GRAIL [36] and WebGestalt [37]. GRAIL [36] identifies relationships amongst genomic disease regions by text mining PubMed abstracts and assessing gene relatedness. WebGestalt [37] performs gene set enrichment analysis given a list of genes or SNPs which it maps to genes using the array specific list of genes. We used the HS and MHS SNP sets to perform our analyses. Because GRAIL accepts a list of SNPs or disease regions and performs its own SNP-to-gene mapping based on LD, we used the gene set generated by GRAIL to perform the remainder of the analyses. Using LD to cluster the SNPs into distinct loci returned similar, but not always, identical results to the naive clustering method used in this study (Additional file 1, **Table S5**). For instance, the SNPs in the MHC locus are all in high LD over long stretches, yet the region is interrupted by multiple recombination hotspots [38]. As a result, the naïve clustering method returns multiple associated loci for the MHC region, while the LD method clumps all the SNPs into one large associated locus. The gene search spaces also differed between the LD method and the *adjacent *mapping implemented for the MHS and HS SNP sets. The average number of genes per locus varies for each disease, ranging from 1 to 27 genes per associated region (data not shown) with the LD approach. As described, the *adjacent *mapping would have at most 4 genes as candidates. Also, the *adjacent *mapping does not restrict the genes to be in LD with the associated locus so for some cases genes at greater distances from the associated region are analysed. An example is two Ephrin receptors (*EFNB2 *Ephrin-B2 a*nd EPHA7 *EPH receptor A7) which *Gentrepid *predicted as candidates for coronary artery disease in the *adjacent *gene set. *Gentrepid *made more predictions for the *adjacent *gene search space than it did for the LD search space for the HS SNP set, but made fewer predictions for the *adjacent *mapping in the MHS SNP set than from the LD (Additional file 1, **Table S6**). This is most likely due to a loss of power as the size of the gene set increases.

**Figure 7:**
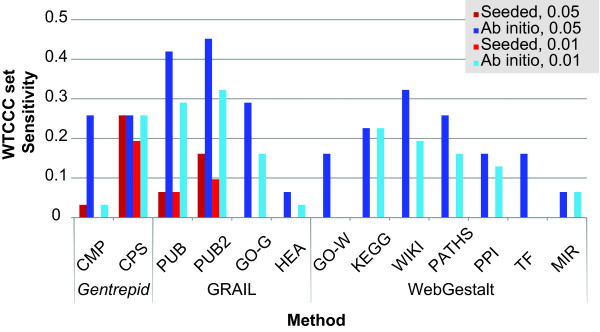
**Comparison of the sensitivity of multiple systems on GWAS data using the LD-gene mapping search space for the MHS set**. CMP and CPS are *Gentrepid *methods. PUB, PUB2, GO-G, and HEA are GRAIL methods. PUB refers to PubMed abstracts up until 2006. PUB2 refers to PubMed abstracts up until 2011. GO-G refers to the gene ontology GRAIL method. HEA refers to the human expression atlas. GO-W, KEGG, WIKI, PATHS, PPI, TF and MIR refer to the methods of WebGestalt. GO-W refers to gene ontology enrichment. KEGG refers to pathway enrichment from the KEGG database. WIKI refers to pathway enrichment from Wikipathways. PATHS refers to pathway enrichment from Pathway Commons. PPI refers to protein interaction enrichment. TF refers to transcription factor enrichment. MIR refers to microRNA enrichment. CPS has the most consistent results.

To compare the available web-based methods, we ran *Gentrepid*, GRAIL and WebGestalt on the HS and MHS sets for each disease using the LD mapped gene search spaces created by GRAIL. We considered a result to be a prediction if the statistical significance of the annotation returned was *p *< 0.05. Overall, WebGestalt and GRAIL returned more candidate gene predictions and more regions with predictions than *Gentrepid *(Additional file 1, **Table S7**). But many of the predictions made by GRAIL and WebGestalt were genes from the same locus, indicating a higher false positive rate. We also calculated the sensitivity, specificity and enrichment ratio of the different methods using the WTCCC candidate genes as the validation set (Additional file 1, **Table S8**) Specificity was high for all the systems, so for more insight into performance we compared the sensitivity using the MHS set (Figure 7). GRAIL had the highest sensitivity with the PubMed abstract method, than when given seeded regions. *Gentrepid *CPS had similar sensitivities for both *ab initio *and *seeded *modes, which remained the same when the *p*-value threshold was lowered (*p *< 0.01). Of note, the sensitivity values are much lower across all the methods using genes selected by linkage disequilibrium compared to the different mapping approaches considered in this study.

## Discussion

GWAS are a valuable approach to identification of loci involved in disease phenotypes. In this work, we developed a method for analysing GWA data that uses a combined statistical and bioinformatic protocol to sort the genotype-phenotype signal from the noise. We adopted a double sift approach, based on genetic and biological knowledge, to identify likely causal genes in selected sets of phenotype-associated SNPs comprising up to 0.2% of genotyped SNPs. This method has revealed hidden information that was missed when the analysis relied solely on the SNPs of highest statistical significance. This may explain some of the missing heritability in GWAS [9]. The biological information employed utilizes pathways and domain-based similarity to find relationships between multiple genes associated with genetic data for specific phenotypes.

### Value of systematic SNP significance investigation

The lower statistical thresholds used in the study capture a large number of phenotype-SNP associations that were not revealed in previous studies. Although lowering the statistical threshold may increase the number of false positive SNPs reported, regions that were otherwise missed by the high significance threshold cut-off adopted in the original study clearly contain phenotype-specific information that departs from random data. The lower statistical significance of these associations may reflect either the rarity of the alleles or those with small effect sizes. Both these types of alleles are believed to be major contributors to the disease phenotypes as the majority of common SNPs associated with the phenotypes studied fail to explain the heritability of many diseased individuals [9]. The replication of known loci in the data of lower significance supports the value of this approach.

### Selecting gene sets associated with each SNP set

Some studies have suggested [10,11], for example those on long range regulation of genes, that the location of controlling elements may be distal to the actual transcripts and protein-coding regions themselves. For this reason, gene sets were selected around SNPs in six different ways to investigate how these SNP to gene selection assumptions affected predictions. The different assumptions, such as the bystander approach, increase the study's gene coverage of the genome, potentially capturing longer range associations between SNPs and genes. Whether these longer range associations are regulatory or arise from linkage disequilibrium remains an open question.

An unavoidable accompaniment of using distance-based gene selection approaches is the introduction of noise into the results which therefore requires stricter filtering. A single associated locus captures a set of at most 4 genes in the proximity-based NN approaches, but in the distance-based BY approaches, some loci that are in gene dense regions link to many more genes. In the largest gene sets tested, on average, there were 16 genes to a 1 Mbp locus. Many of the predictions made by *Gentrepid *are for the largest loci: 1 Mbp BY, 0.5 Mbp BY and *adjacent *NN. In many instances the predicted gene is not the nearest gene to the implicated SNP (Table 2). This may truly demonstrate long range regulatory effects, or alternatively the inclusion of more genes may simply increase the chances of predictions. The most successful approaches, as judged by the enrichment ratios, specificity and sensitivity measurements on the validation sets, are the *adjacent *and 0.1 Mbp approaches. Both these sets have similar search space sizes and contain less noise than the larger BY sets. However, there may be an element of self-selection in this result, if geneticists typically only scan the immediate vicinity of the locus studied for the disease gene. Even so, the more generous SNP/gene assumptions did not unduly lower the performance of the system, with multiple instances of consensus amongst predictions across the different sets [39]. Enrichment ratios varied from 15 to 25 depending on the thresholds. For the maximal search space of 3000 genes, this is roughly equivalent to 120-200 disease candidates per phenotype: a number that is feasible to scan with current sequencing technologies.

### Performance in *seeded *versus *ab initio *mode

Predictions based on known disease genes are inherently limited by what is already known about a phenotype. In *seeded *mode, *Gentrepid *is an effective tool to assist in the discovery of phenotype-related genes in novel loci. The *ab initio *methodology is a powerful discovery tool for finding novel genotype-phenotype relationships for complex diseases. For diseases with Mendelian inheritance, *ab initio *mode is also likely to be advantageous if only a small percentage of cases arise from known disease genes.

CPS *seeded *mode is generally a more powerful discovery tool when retrieving novel genes associated with pathways involving disease genes previously linked to the phenotype. In this mode, the candidate gene search space is enriched for known disease pathways, increasing the chances of retrieving genes that share this pathway. In contrast, *ab initio *mode only considers genes within the candidate loci and excludes many of the confirmed disease genes: a more agnostic approach which may be informative.

In addition to the constraints described above for *ab initio *predictions, the success of *Gentrepid *predictions using known disease genes depends on how informative these genes are for the phenotype. A number of factors influence the system's ability to make predictions including the quality of the input GWA data for the specific phenotype, and genome coverage of pathways specific to phenotype. Even when the entire genome is considered, only 57% of characterized genes have *Gentrepid *annotations and are thus potentially predictable as candidates. Most of this coverage is due to Pfam domains, while pathways cover up to 20% of annotated genes (Figure 2). Thus the system is unable to make predictions for around 40% of the genome which may or may not be associated with the phenotype. Outside these phenotype-specific constraints, a threshold number of loci are required for the prediction to be significant. As discussed, generic pathways that are not highly informative or specific, increase the chances of random predictions by CPS, as do common domains by CMP (e.g. Ras PF00071).

### Performance of CMP versus CPS

In our previous benchmark, which used a dataset of Mendelian diseases developed by Turner *et al *[40], we found CPS more effective in retrieving candidates. Using GWA data for complex diseases, the domain-based CMP module of *Gentrepid *made many plausible predictions. The auto-detected domain comparison in CMP removes the need to rely on the current annotations of human proteins, which are still lacking [41]; or on whole gene sequence-similarity which is less accurate [42]. However it was interesting that CPS was still more effective in replicating known disease genes including some loci where a disease gene has not previously been allocated. Of the 29 known disease genes, 16 were predicted by CPS. The predictions made by CMP may be spurious, although the random simulations suggest otherwise. Alternatively, the superior performance of CPS in replicating known disease genes may be a selection effect. The genes within the sets were determined to be disease causing based on known disease pathways and interactions. Specifically, geneticists may have been searching for disease genes for complex diseases based on experience gained from Mendelian diseases. In the case of Mendelian diseases, this approach may apply, as penetrance is high for monogenic disorders. Overlapping functionality arising from similar domain structures would not be a very strong predictor for Mendelian diseases, as genes with similar functions would not have highly penetrant phenotypes. Genes with overlapping functions may mask each other's defects, be insufficient to cause the disease alone, and perhaps only fractionally increase the risk. In complex or polygenic disorders, genes with overlapping functions that are mutated or dysregulated may be more common, and hence predictions by CMP may be more suited for gene discovery in these disease states.

### Performance on validation sets

*Gentrepid *was capable of replicating genes already implicated by past genetic studies and the WTCCC GWAS. For loci flagged by the GWA study that were previously noted in OMIM, CPS successfully prioritised the known disease genes. For the genes determined by the WTCCC as likely candidates, either CPS or CMP was capable of predicting the candidates. As the two sets were generated from different genetic sources, it is not unusual for the system to perform differently on both. The known disease genes were determined through family linkage analysis studies, but the WTCCC gene validation set was generated from SNPs that are population based. It may be that the known disease genes are family specific or "private" and were not in the population studied by the WTCCC. The WTCCC candidates were selected by looking at the nearest genes, and not further, possibly missing other real candidates. Therefore differentiating false positives from true positives is almost subjective.

Although the data were averaged across the seven WTCCC phenotypes for this analysis, the performance of the system is somewhat phenotype dependent. The stringency threshold on some phenotypes has to be lower to compensate for genetic heterogeneity in diseases such as hypertension. For the autoimmune diseases where highly significant results are within gene dense regions such as the MHC locus or the cytokine cluster on human chromosome 5, the identification of the causal gene cannot be resolved through data mining analyses as all genes in the region share similar functions and protein structures. However the system does give some important information in these instances by identifying the domain or pathway that is being represented by the gene clusters, which in the previous example are genes regulating immunity. It is also important therefore to use all the genes within the cluster for analysis, and hence the SNP/gene distance based approach, as less common pathways within the cluster may be important in the multiple loci analyses that the system performs. The genetic and biological complexity of the diseases is demonstrated in the raw data from the GWA SNP analysis. To decipher this complexity, the biomolecular and protein analysis automatically detects commonality between multiple loci detected and thus, to some extent, compensates for current statistical genetic methods used on GWA data that test each SNP is isolation from the others.

### Comparison to other systems

Each method studied here has its strengths and weaknesses which should be kept in mind during use. For instance, WebGestalt [37] looks for gene annotation enrichment but does not take into account gene duplication found within the same locus. Gene clusters of similar genes such as those in the MHC locus are given equal weight as those from multiple regions and would inflate particular results. GRAIL [36] and *Gentrepid *on the other hand, correct for this by adjusting the calculations so genes from the same associated region are not counted multiple times. Many of the GRAIL predictions were made through the text of recent PubMed abstracts, while fewer significant predictions were made with the text prior to GWAS publications, indicating that the results are mostly returning what we know and few *de novo *candidates. In short, GRAIL is acting as a retrieval tool. *Gentrepid *appears to be predicting novel candidates with its functional domain-based approach. Also, very few of the predictions overlapped between the different data sources used in each system. This emphasizes that one source of data may not be sufficient to make candidate gene predictions and that using alternate tools and data is wise, although these need to be carefully tested and understood in isolation.

Several advantages of *Gentrepid *are: it allows analysis of large datasets such as the MWS and WS set used in this study; like GRAIL, *Gentrepid *allows users the flexibility to enter genes or genome intervals, but uses different data sources for predictions. *Gentrepid *performs similar enrichment analysis to WebGestalt but accounts for multiple genes implicated from the same region.

## Conclusions

In conclusion, we performed an extensive analysis of the *Gentrepid *system using GWA data. The approach used four sets of significant SNPs at different significance thresholds. SNPs were mapped to the genome in six different ways and the resulting search spaces analysed with the *Gentrepid *candidate gene prediction system. The results show that using what is known about the disease (*seeded*) as well as a blind approach (*ab initio) *is beneficial in the discovery and prediction of candidate disease genes. Further to this, using a less stringent SNP association threshold allows true signals to be detected which can be filtered using biomolecular information. Also, when using gene selection approaches which include genes that are not the nearest gene to the implicated SNP, *Gentrepid *makes significant predictions without unduly lowering the performance of the system. As the predictions remain dependent on what we already know in the protein knowledge base and on disease information, the system is only as good as the underlying databases. Further detailed work on discovery and annotation is required to take advantage of the existing GWA data. We believe this method to be an important tool in analysing GWAS as current methods are less flexible and require more data processing.

## Methods

### WTCCC data

We obtained SNP data from the WTCCC [33] case-control studies of seven diseases: bipolar disorder (BD), coronary artery disease (CAD), Crohn's disease (CD), hypertension (HT), rheumatoid arthritis (RA), type I diabetes (T1D) and type II diabetes (T2D). The WTCCC GWAS used the Affymetrix chip set with approximately 500,000 known SNPs (Affy500k), with SNP positions referenced to the human genome sequence assembly from NCBI (build 35). We mapped these SNPs to 489,763 autosomal SNPs on the genome assembly (build 36.3), and 459,231 SNPs following WTCCC quality control [33].

### OMIM known disease genes

We extracted known disease genes and loci from the OMIM database [35] Morbid Map flat file by performing a text search for the disease name or parts thereof. These were then manually filtered for relevant loci.

### Choice of SNP significance thresholds

An initial set of associated SNPs was filtered from the summary data of SNPTEST [43], a program that performs a series of association tests on the genotypes obtained from the case-control studies. The *p*-value of the trend test statistic (Cochran-Armitage test) [44] of the additive genetic model was used as a test statistic for SNP significance. The levels of significance chosen as the SNP association thresholds were determined using quantile-quantile (Q-Q) plots of the datasets (Additional file 2, **Figure S1**). The Q-Q plots were constructed by plotting the observed -log_10_(*p_GWA_*) of the SNPs against the expected -log_10_(*p_GWA_*), constructed under the null hypothesis that there is no association between the SNPs and the phenotype. Visual inspection of the Q-Q plots shows the distribution of test statistics of the observed SNPs for each phenotype begins to deviate from the expected distribution under the null hypothesis near *p_GWA _*≈10^-2 ^and is distinctly different at *p_GWA _*≤ 10^-3^.

Four different *p*-value thresholds were used to create four associated SNP data sets for each phenotype: a weakly significant set (WS, *p_GWA _*≤ 10^-3^), a moderately-weak significant set (MWS, *p_GWA _*≤ 10^-4^), a moderately-high significant set (MHS, *P_GWA _*≤ 10^-5^), and a highly significant SNP set (HS, *p_GWA _*< 5 × 10^-7^). The final HS set is equivalent to the threshold used in the WTCCC study, where the *p*-value was determined based on the *a priori *probability of association, and not the typical multiple-comparison or Bonferroni correction [33].

SNPs within the sets were clustered based on physical distance to one another through a naïve distance-based clustering process: a SNP within 50 Kbp of another SNP was considered to form a cluster. This value was chosen based on the average size of haplotype blocks [45].

### Construction of candidate gene search spaces

We used *Gentrepid *to predict and prioritise candidate disease genes selected from phenotype-associated gene sets generated from the SNP loci. Gene sets were constructed using one of two major assumptions: disease-associated SNPs are either resident in, or adjacent to, the disease gene; or the disease-associated SNPs may be near, but not closest or adjacent to, the disease gene. The first assumption we termed the nearest neighbour gene selection approach (NN) and the second assumption the bystander approach (BY).

For the NN approach, three sets of genes were created: a set containing genes with SNPs internal to the gene boundary defined by RefSeq [26], termed the *resident *set; a second set with SNPs resident in the gene or directly adjacent to it, termed the *nearest *set; and a third set with the SNPs either resident in, or directly adjacent to, the four nearest genes, termed the *adjacent *set. The *nearest *set corresponds to the set commonly selected by nearest neighbour approaches in most recent GWAS [3]. In the *adjacent *set, genes on both strands of the chromosome were considered in both the 5' and 3' direction. For both the *nearest *and *adjacent *sets the physical distance between the SNP and the gene was not used as a constraint.

For the BY approach, three intervals of different sizes were tested. These values were chosen based on average distances of transcriptional regulatory elements from the genes they control [46,47]. Genes on both strands around each of the SNPs were pooled from flanking intervals of 0.1 Mbp, 0.5 Mbp or 1 Mbp in width.

SNP and gene density are non-uniform across the genome and gene sizes vary, all of which influence the number of positional gene candidates available for analysis. To test for bias due to SNP coverage by the Affymetrix chip set, we first checked the SNP distribution across the genome. SNP positions and the frequency of SNPs in different gene regions (exonic, intronic, UTRs) and intergenic locations of the genome were calculated by creating density plots. To determine if gene coverage was affected by the various SNP-to-gene search space construction assumptions, we calculated the percentage of genes in the genome covered by SNPs on the Affy500K chip set using each approach. We also wished to determine if these genes were represented in *Gentrepid *by associated pathways and domains.

### Prediction and prioritisation of candidate genes

To determine which SNPs are more likely to contribute to the disease phenotype, a set of analyses were performed using direct SQL queries of the in-house *Gentrepid *database https://www.gentrepid.org. *Gentrepid*'s two modes of input, *seeded *mode and *ab initio *mode were used to determine the common properties of phenotype-specific genes. *Seeded *mode is assisted by phenotype-associated genes from OMIM as seeds (Additional file 1, **Table S1**)  *Ab initio *mode uses only genes pooled from the search spaces.

Genes in each data set were prioritised based on phenotype-associated common pathways (via CPS) and common domains (via CMP). In previous work using CPS, pathways containing at least two genes from distinct loci were ranked based on the total number of loci involved, as described in George *et al *[16]. This is not entirely satisfactory because it favours large pathways. For instance, a pathway containing a large number of genes may be selected over a more pertinent smaller pathway or a subnet. To test the likelihood of a pathway being associated with a phenotype, genes were bi-partitioned based on whether they were associated with (a) the phenotype in question and (b) the pathway in question. To calculate the significance, Fisher's exact test was performed using the *fisher.test *function in R http://www.r-project.org/[48]. For CPS *seeded *and *ab initio*, gene predictions were filtered based on the statistical significance of the pathway using a threshold of *p_path _*< 0.05, and prioritised based on the lowest *p*-value score of the pathway they shared.

For CMP, the domains of phenotype-specific genes were queried from the database and compared to domains of other phenotype-specific genes in the data set (*ab initio) *or domains of known disease genes (seeded), as described in George *et al *[16]. For CMP *seeded*, predictions are based on a pair wise similarity score between the candidate and a known disease gene between 0 (no similarity) and 1 (identical) [16]. Using a benchmark set of oligogenic diseases with Mendelian inheritance suggested by Turner *et al *[40], we previously determined that a pair wise similarity score of 0.4 between the test gene and the known disease gene is a conservative threshold above which the test gene can be considered a candidate [16]. Results above the threshold score of 0.4 were filtered and prioritised. In CMP *ab initio *mode, the domain combination was tested for over-representation in the constructed intervals compared to the genome as a whole through upper and lower significance tests, based on a range of expected values relating to domain correlation within genes. The expected number of domains was calculated based on the value of *p*, representing the extrema of the level of correlation between domains in genes (*p_min_*, *p_max_*), and more specifically the likelihood of occurrence of the domain combination by chance [16]. Within a gene, domain duplications are reasonably common [25] leading to an anomalously low *p_max_*. Thus a revised *p_max _*which ignores multiple copies of domains was calculated to correct for this effect (*p_max_unique_*). The gene predictions were filtered on significance based on the three χ^2 ^tests (χ^2^*_min_*, χ^2^*_max_unique _*and χ^2^*_max)_*. A χ^2 ^value greater than 7.88 is significant at the 0.005 level, but we adopted more conservative values of χ^2^*_max_unique _*≥ 10^5 ^for multidomain proteins, and χ^2 min ^≥ 10^2 ^for single domain proteins. The predictions were then filtered against random simulations described below to remove false positives. Finally, the three χ^2 ^scores were correlated with the random predictions using the Spearman's rank correlation coefficient test to choose the best metric for prioritisation. Based on this test χ^2 min ^was chosen.

As a control we selected random SNP sets of similar size to the phenotype-specific data and mapped these to genes using the same protocols described above (NN, BY). The number of SNPs selected for each search space was similar to the number of clusters formed by the SNPs in the WTCCC data, and not the exact number of significant SNPs. This was done to account for clusters in the phenotype-specific data due to linkage disequilibrium or SNP-disease association. For each random set, we ran *Gentrepid *CPS and CMP in both *seeded *and *ab initio *modes, and tracked predictions and significance scores. The randomization results were averaged across 1000 replications.

### Validation of predictions and metrics

We took several approaches to assess the ability of the two *Gentrepid *modules to extract positional candidates. Firstly, we studied the ability of *Gentrepid *to extract and prioritise known disease genes and loci from the phenotype data. When known disease genes were employed as seeds for *Gentrepid *predictions, a leave-one-out cross validation technique was used. In this process, known disease genes were iteratively removed as seeds during the prediction process, and the resulting rank of the withheld gene was then assessed. The ranks of known gene predictions in *ab initio *mode were also calculated. Secondly, we assessed the *Gentrepid *results against genes associated with the HS SNPs by the WTCCC. Finally, predictions on the GWA-implicated loci were compared to predictions made by *Gentrepid *on the random data for the chosen levels of stringency using both the NN and BY gene selection assumptions. We calculated the specificity, sensitivity and enrichment ratios for each of the validation sets as described in our previous work [17] and also plotted ROC curves for an overall comparison of the system (Additional file 3, **Figure S2**).

### Comparison to other online methods

We selected GRAIL [36] and WebGestalt [37] as online tools for comparison because they both take input from GWAS data, and perform gene annotation enrichment analysis similar to *Gentrepid*. GRAIL makes three independent predictions using three different sources of data: text mining of PubMed abstracts, Gene Ontology (GO) annotations [49] or mRNA expression levels from The Novartis Gene Expression Atlas [50]. Genes are given a significance score [36]. WebGestalt performs gene enrichment analysis on a user-defined gene/protein list or a list of SNPs from typical GWAS arrays such as those from Affymetrix or Illumina. The analysis is performed by searching for enrichment of gene annotations from GO, pathways from KEGG, Wikipathways and Pathway commons, transcription factor binding site motifs and microRNA target enrichment. Genes are not individually ranked, but a *p*-value is calculated for every annotation returned as a result [37].

To use a standard set of genes across all three tools, we had to utilize the GRAIL gene search space as it was the most restrictive. The GRAIL gene search space is generated automatically from a user-defined list of SNPs according to the linkage disequilibrium of the associated locus. For each disease, we uploaded the list of associated significant SNPs for the HS and MHS set. GRAIL performs LD mapping and returns a list of query genes. This LD gene list was then used as input for both *Gentrepid *and WebGestalt.

We ran GRAIL using the PubMed Text 2006 and 2011, GO 2006 and Novartis databases. A seeded search can also be performed in GRAIL by including genomic regions known to be associated with the disease. We used the OMIM set defined earlier as the known gene set and ran queries on the same databases. For each gene in the search space, we stored the prediction *p*-values for each data source.

We ran WebGestalt enrichment analysis on the same gene lists analyzed by GRAIL. We used the "hspaiens_genome" as the reference gene list for all enrichment analyses and ran the web tool using the default settings, except for the significance levels which were set to *p *< 0.05 (default of Top 10). We performed GO analysis, KEGG analysis, Wikipathways analysis, Pathway Commons analysis, transcription factor target analysis, microRNA target analysis, and protein interaction network module analysis. For each gene, we stored the most significant *p*-value for each analysis. We considered two sets of predictions using thresholds significance *p*-values of either 0.05 or 0.01.

We ran *Gentrepid *on the LD-mapped gene search space, using both CPS and CMP in *seeded *and *ab initio *mode. We stored the CPS *p*-values as the prediction *p*-values. CMP scores were evaluated at scores of either 0.4 or 0.8 for *seeded *CMP, and χ^2 min ^scores of either 100 or 10^5 ^for *ab initio *CMP.

We then compared the *p*-values of the genes across all the systems. We also calculated the specificity, sensitivity and enrichment ratios on the WTCCC validation set and compared across the different methods.

## List of abbreviations

GWAS: Genome-wide association studies; WTCCC: Wellcome Trust Case-Control Consortium; CPS: common pathway scanning; CMP: common module profiling; BD: Bipolar disorder; CAD: Coronary artery disease; CD: Crohn's disease; HT: Hypertension; RA: Rheumatoid arthritis; T1D: Type I diabetes; T2D: Type II diabetes; NN: Nearest neighbour approach; BY: Bystander approach; WS: Weakly significant set; MWS: Moderately-weak significant set; MHS: moderately-high significant set; HS: highly significant set.

## Competing interests

One of the authors (MAW) has a US patent application on the CMP methodology.

## Authors' contributions

SB carried out the data mining and analysis, and worked on the design of the project and algorithms. JL worked on the algorithms. MO worked on the CMP predictions. BG, DF and MB participated in the design of the study. MAW conceived the study, participated in its design, helped draft the manuscript and reviewed the results from the data analysis. All authors read and approved the final manuscript.

## Supplementary Material

Additional file 1 Gentrepid validation gene sets and additional benchmarking results.
Table S1 OMIM phenotype associated genes used as seeds for the *seeded *mode and as the known disease gene validation set.
Table S2 Genes included in the known validation set.
Table S3 Genes included in the WTCCC validation set.
Table S4 Specificity, Sensitivity and Enrichment ratios for validation sets across all phenotypes.
Table S5 LD versus naïve clustering.
Table S6 Comparison of the number of significant *Gentrepid *predictions between LD and adjacent gene selection sets.
Table S7 Total numbers of significant predictions across *Gentrepid*, GRAIL and WebGestalt.
Table S8 Specificity, Sensitivity and Enrichment ratios for WTCCC validation set for *Gentrepid*, GRAIL and WebGestalt. Click here for file

Additional file 2 **Figure S1 Q-Q plots of expected values of the associated trend test *p*-values versus observed generated for each phenotype in black and uniform distribution in grey**. Click here for file

Additional file 3 **Figure S2 ROC curves for *Gentrepid *on known and WTCCC validation sets**. CPS is represented by the dashed lines, CMP by the filled lines. The colors indicate the SNP-to-gene mapping set used. The first column from the left are the results for the known validation set using *seeded *mode, The second column are the known validation set under *ab initio*. The third column is the WTCCC validation set *seeded *results. And the fourth column the WTCCC set, *ab initio*. The top panels are the HS sets. The next set of panels the MHS set, the third MWS and the bottom panels the WS set. The grey line in each plot represents what a random guess should give. CPS is above the line for most cases. CMP is below. CPS with the 0.1 Mbp or *adjacent *set performs the best. Click here for file
