# Supplementary material for: Analysis of genome-wide association study data using the protein knowledge base
Source: BMC Genet. 2011 Nov 13;12:98. doi: 10.1186/1471-2156-12-98 (PMC3261104; doi:10.1186/1471-2156-12-98)
Supplement: Additional file 1 — Gentrepid validation gene sets and additional benchmarking results. Table S1 OMIM phenotype associated genes used as seeds for the seeded mode and as the known disease gene validation set. Table S2 Genes included in the known validation set. Table S3 Genes included in the WTCCC validation set. Table S4 Specificity, Sensitivity and Enrichment ratios for validation sets across all phenotypes. Table S5 LD versus naïve clustering. Table S6 Comparison of the number of significant Gentrepid predictions between LD and adjacent gene selection sets. Table S7 Total numbers of significant predictions across Gentrepid, GRAIL and WebGestalt. Table S8 Specificity, Sensitivity and Enrichment ratios for WTCCC validation set for Gentrepid, GRAIL and WebGestalt. [file 1471-2156-12-98-S1.DOCX]

**Table S1 OMIM phenotype associated genes used as seeds for the *seeded* disease gene approach**

| **Disease** | **Genes (HUGO)** | **Gene Entrez IDs** | **OMIM IDs** |
| --- | --- | --- | --- |
| Bipolar Disorder (BD) | *SLC6A3, XBP1, FKBP5, and HTR2A* | 6531, 7494, 2289, 3356 | 125480, 612371, 608516 |
| Coronary Artery Disease (CAD) | *ABCA1, MEF2A, LRP6, CCL2, CX3CR1, LPA, IRS1, KL, PON1, PON2, MMP3, CD36, and NOS3* | 19, 4205, 4040, 6347, 1524, 4018, 3667, 9365, 5444, 5445, 4314, 948, 4846 | 143890, 147545, 152200, 158105, 168820, 185250, 601470, 602447, 603507, 604824, 608320, 610938 |
| Crohn’s Disease (CD) | *IL23R, DEFB4, DLG5, CARD15, and IL6* | 149233, 1673, 9231, 64127, 3569 | 612261, 266600 |
| Hypertension (HT) | *HSD11B2, NR3C2, PNMT, AGTR1, PTGIS, NPR3, BMPR2, ACSM3, KCNMB1, ADD1, AGT, ECE1, GNB3, RETN, NOS3, NOS2A, CYP3A5, CYP11B2, CPS1, SELE, ATP1B1, RGS5, and EPHX1* | 3291, 4306, 5409, 185, 5740, 4883, 659, 6296, 3779, 118, 183, 1889, 2784, 56729, 4846, 4843, 1577, 1585, 1373, 6401, 481, 8490, 2052 | 145500, 108962, 124080, 125853, 145505, 178600, 189800, 218030, 265380, 605115, 608622 |
| Rheumatoid Arthritis (RA) | *STAT4, IL10, CD244, HLA-DRB1, CIITA, NFKBIL1, PADI4, PTPN22, RUNX1, SLC22A4, MIF, and IL6* | 6775, 3586, 51744, 3123, 4261, 4795, 23569, 26191, 861, 6583, 4282, 3569 | 180300, 604302 |
| Type I Diabetes (T1D) | *IL6, TCF1, OAS1, FOXP3, ITPR3, PTPN22, IL2RA, CTLA4, CCR5 and SUMO4* | 3569, 6927, 4938, 50943, 3710, 26191, 3559, 1493, 1234, 387082 | 222100, 612522, 600320, 601388, 601942 |
| Type II Diabetes (T2D) | *PTF1A, TCF7L2, KCNJ11, ABCC8, MAPK8IP1, UCP3, TCF1, IPF1, IRS2, LIPC, SLC2A4, TCF2, RETN, AKT2, GPD2, NEUROD1, IRS1, CAPN10, PTPN1, PPARG, SLC2A2, IGF2BP2, WFS1, CDKAL1, ENPP1, IL6, GCK, PAX4, SLC30A8, and HNF4A* | 256297, 6934, 3767, 6833, 9479, 7352, 6927, 3651, 8660, 3990, 6517, 6928, 56729, 208, 2820, 4760, 3667, 11132, 5770, 5468, 6514, 10644, 7466, 54901, 5167, 3569, 2645, 5078, 169026, 3172 | 125853, 125851, 601283, 609069, 601665, 138160 |

**Table S2 Genes included in the known OMIM validation set**

| **Disease** | **Genes (HUGO)** | **Gene Entrez IDs** | **Search space set** | | | | | | **Significance level** | | | |
| --- | --- | --- | --- | --- | --- | --- | --- | --- | --- | --- | --- | --- |
|  |  |  | **1M** | **0.5M** | **0.1M** | **A** | **N** | **R** | **HS** | **MHS** | **MWS** | **WS** |
| CAD | *CX3CR1* | 1524 | x | x |  | x |  |  |  |  | x | x |
|  | *IRS1* | 3667 | x |  |  | x |  |  |  |  | x | x |
|  | *LPA* | 4018 | x | x |  |  |  |  |  |  |  | x |
|  | *LRP6* | 4040 | x |  |  |  |  |  |  |  |  | x |
|  | *NOS3* | 4846 | x | x |  |  |  |  |  |  |  | x |
|  | *CD36* | 948 | x | x |  | x |  |  |  |  | x | x |
| CD | *IL23R* | 149233 | x | x | x | x | x | x | x | x | x | x |
|  | *CARD15* | 64127 | x | x | x | x | x | x | x | x | x | x |
|  | *DLG5* | 9231 | x | x | x | x | x | x |  |  |  | x |
| HT | *AGT* | 183 | x | x | x | x |  |  |  |  |  | x |
|  | *AGTR1* | 185 | x | x |  |  |  |  |  |  |  | x |
|  | *EPHX1* | 2052 | x |  |  |  |  |  |  |  |  | x |
|  | *PTGIS* | 5740 | x | x |  |  |  |  |  |  |  | x |
| RA | *PTPN22* | 26191 | x | x | x | x |  |  | x | x | x | x |
|  | *HLA-DRB1* | 3123 | x | x | x | x |  |  | x | x | x | x |
|  | *IL10* | 3586 | x | x |  | x |  |  |  |  |  | x |
|  | *CIITA* | 4261 | x |  |  |  |  |  |  |  |  | x |
|  | *NFKBIL1* | 4795 | x | x |  |  |  |  |  |  | x | x |
| T1D | *CCR5* | 1234 | x | x |  |  |  |  |  |  | x | x |
|  | *CTLA4* | 1493 | x | x | x | x | x |  |  |  | x | x |
|  | *PTPN22* | 26191 | x | x | x | x |  |  | x | x | x | x |
|  | *IL2RA* | 3559 | x | x | x | x | x | x |  | x | x | x |
|  | *ITPR3* | 3710 | x | x | x | x | x | x | x | x | x | x |
|  | *OAS1* | 4938 | x |  |  |  |  |  |  | x | x | x |
| T2D | *AKT2* | 208 | x |  |  |  |  |  |  |  |  | x |
|  | *CDKAL1* | 54901 | x | x | x | x | x | x |  | x | x | x |
|  | *TCF2* | 6928 | x | x |  |  |  |  |  |  |  | x |
|  | *TCF7L2* | 6934 | x | x | x | x | x | x | x | x | x | x |
|  | *WFS1* | 7466 | x |  |  |  |  |  |  |  |  | x |

Abbreviations: 1M, 1Mbp SNP to gene mapping; 0.5M, 0.5Mbp; 0.1M, 0.1Mbp; A, Adjacent; N,Nearest; R, Resident; HS, highly significant; MHS, moderately-high significance, MWS: moderately-weak significance WS: weakly significant.

**Table S3 Genes included in the WTCCC validation set**

| **Disease** | **Genes (HUGO)** | **Gene Entrez IDs** | **Search space set** | | | | | | **Significance level** | | | |
| --- | --- | --- | --- | --- | --- | --- | --- | --- | --- | --- | --- | --- |
|  |  |  | **1M** | **0.5M** | **0.1M** | **A** | **N** | **R** | **HS** | **MHS** | **MWS** | **WS** |
| BD | 84516 | *DCTN5* | x | x | x | x |  |  |  |  |  | x |
|  | 79728 | *FLJ21816* | x | x | x | x | x | x |  |  |  | x |
|  | 2560 | *GABRB1* | x | x | x | x | x | x |  |  | x | x |
|  | 4706 | *NDUFAB1* | x | x | x |  |  |  |  |  |  | x |
|  | 8224 | *SYN3* | x | x | x | x | x | x |  |  | x | x |
| CAD | 11173 | *ADAMTS7* | x | x | x | x | x | x |  |  | x | x |
|  | 1029 | *CDKN2A* | x | x | x |  |  |  | x | x | x | x |
|  | 1030 | *CDKN2B* | x | x | x | x | x |  | x | x | x | x |
|  | 4507 | *MTAP* | x | x |  | x |  |  | x | x | x | x |
|  | 25902 | *MTHFD1L* | x | x | x | x | x | x |  | x | x | x |
| CD | 55054 | *ATG16L1* | x | x | x | x | x | x | x | x | x | x |
|  | 8927 | *BSN* | x | x | x | x | x | x |  | x | x | x |
|  | 64127 | *CARD15* | x | x | x | x | x | x | x | x | x | x |
|  | 149233 | *IL23R* | x | x | x | x | x | x | x | x | x | x |
|  | 345611 | *IRGM* | x | x | x | x | x |  | x | x | x | x |
|  | 4485 | *MST1* | x | x | x | x |  |  |  | x | x | x |
|  | 159296 | *NKX2-3* | x | x | x | x | x |  | x | x | x | x |
|  | 5771 | *PTPN2* | x | x | x | x | x | x | x | x | x | x |
|  | 6774 | *STAT3* | x | x | x | x | x | x |  |  | x | x |
|  | 7128 | *TNFAIP3* | x | x |  | x |  |  |  | x | x | x |
|  | 22891 | *ZNF365* | x | x | x | x | x | x | x | x | x | x |
| HT | 1131 | *CHRM3* | x |  |  |  |  |  |  |  | x | x |
| RA | 3002 | *GZMB* | x | x |  | x |  |  |  |  | x | x |
|  | 3123 | *HLA-DRB1* | x | x | x | x |  |  | x | x | x | x |
|  | 3559 | *IL2RA* | x | x | x | x | x | x |  | x | x | x |
|  | 3560 | *IL2RB* | x | x | x | x | x | x |  | x | x | x |
|  | 81621 | *KAZALD1* | x | x | x | x |  |  |  |  |  | x |
|  | 5588 | *PRKCQ* | x | x |  | x | x |  |  | x | x | x |
|  | 26191 | *PTPN22* | x | x | x | x |  |  | x | x | x | x |
| T1D | 969 | *CD69* | x | x | x | x | x | x |  |  |  | x |
|  | 1493 | *CTLA4* | x | x | x | x | x |  |  |  | x | x |
|  | 28955 | *DEXI* | x | x | x | x |  |  | x | x | x | x |
|  | 2065 | *ERBB3* | x | x | x | x | x | x | x | x | x | x |
|  | 3123 | *HLA-DRB1* | x | x | x | x |  |  | x | x | x | x |
|  | 3559 | *IL2RA* | x | x | x | x | x | x |  | x | x | x |
|  | 23274 | *KIAA0350* | x | x | x | x | x | x | x | x | x | x |
|  | 5781 | *PTPN11* | x | x | x | x | x | x | x | x | x | x |
|  | 5771 | *PTPN2* | x | x | x | x | x | x |  | x | x | x |
|  | 26191 | *PTPN22* | x | x | x | x |  |  | x | x | x | x |
|  | 10019 | *SH2B3* | x | x | x | x | x | x | x | x | x | x |
|  | 10906 | *TRAFD1* | x | x | x | x | x | x | x | x | x | x |
| T2D | 54901 | *CDKAL1* | x | x | x | x | x | x |  | x | x | x |
|  | 1029 | *CDKN2A* | x | x | x |  |  |  |  |  |  | x |
|  | 1030 | *CDKN2B* | x | x | x | x | x |  |  |  |  | x |
|  | 79068 | *FTO* | x | x | x | x | x | x | x | x | x | x |
|  | 3087 | *HHEX* | x | x | x | x | x |  |  | x | x | x |
|  | 6934 | *TCF7L2* | x | x | x | x | x | x | x | x | x | x |

Abbreviations: 1M, 1Mbp SNP to gene mapping; 0.5M, 0.5Mbp; 0.1M, 0.1Mbp; A, Adjacent; N,Nearest; R, Resident; HS, highly significant; MHS, moderately-high significance, MWS: moderately-weak significance WS: weakly significant.

**Table S4 Specificity, Sensitivity and Enrichment ratios for both validation sets across all phenotypes**

| **Threshold** | **Validation set** | **SNP/gene** | **CPS-s** | | | **CPS-ab** | | | **CPS** | | | **CMP-s** | | | **CMP-ab** | | | **CMP** | | | ***Seeded*** | | | ***Ab initio*** | | | **Total** | | |
| --- | --- | --- | --- | --- | --- | --- | --- | --- | --- | --- | --- | --- | --- | --- | --- | --- | --- | --- | --- | --- | --- | --- | --- | --- | --- | --- | --- | --- | --- |
| **WS** |  |  | **Sp** | **Se** | **ER** | **Sp** | **Se** | **ER** | **Sp** | **Se** | **ER** | **Sp** | **Se** | **ER** | **Sp** | **Se** | **ER** | **Sp** | **Se** | **ER** | **Sp** | **Se** | **ER** | **Sp** | **Se** | **ER** | **Sp** | **Se** | **ER** |
|  | K | 1Mbp | 0.98 | 0.36 | 19.78 | 0.94 | 0.40 | 6.52 | 0.94 | 0.40 | 6.52 | 0.99 | 0.04 | 4.22 | 1.00 | 0.00 | 0.00 | 0.99 | 0.04 | 3.04 | 0.97 | 0.40 | 15.08 | 0.94 | 0.40 | 6.21 | 0.93 | 0.44 | 6.06 |
|  |  | 0.5Mbp | 0.99 | 0.35 | 24.29 | 0.96 | 0.40 | 9.09 | 0.96 | 0.40 | 9.06 | 0.99 | 0.05 | 5.46 | 1.00 | 0.00 | 0.00 | 0.99 | 0.05 | 3.95 | 0.98 | 0.40 | 17.46 | 0.95 | 0.40 | 8.47 | 0.94 | 0.45 | 8.05 |
|  |  | 0.1Mbp | 0.97 | 0.36 | 10.71 | 0.89 | 0.45 | 3.95 | 0.89 | 0.45 | 3.95 | 0.99 | 0.00 | 0.00 | 0.99 | 0.00 | 0.00 | 0.98 | 0.00 | 0.00 | 0.96 | 0.36 | 9.32 | 0.88 | 0.45 | 3.65 | 0.87 | 0.45 | 3.54 |
|  |  | Adjacent | 0.97 | 0.40 | 12.31 | 0.90 | 0.47 | 4.44 | 0.90 | 0.47 | 4.44 | 0.99 | 0.00 | 0.00 | 1.00 | 0.00 | 0.00 | 0.99 | 0.00 | 0.00 | 0.96 | 0.40 | 9.81 | 0.89 | 0.47 | 4.27 | 0.88 | 0.47 | 3.99 |
|  |  | Nearest | 0.92 | 0.50 | 6.31 | 0.79 | 0.67 | 3.17 | 0.79 | 0.67 | 3.17 | 0.99 | 0.00 | 0.00 | 0.98 | 0.00 | 0.00 | 0.97 | 0.00 | 0.00 | 0.91 | 0.50 | 5.48 | 0.78 | 0.67 | 3.02 | 0.77 | 0.67 | 2.90 |
|  |  | Resident | 0.94 | 0.40 | 6.54 | 0.85 | 0.40 | 2.68 | 0.85 | 0.40 | 2.68 | 0.99 | 0.00 | 0.00 | 0.93 | 0.00 | 0.00 | 0.93 | 0.00 | 0.00 | 0.93 | 0.40 | 5.74 | 0.79 | 0.40 | 1.91 | 0.79 | 0.40 | 1.89 |
|  | W | 1Mbp | 0.98 | 0.14 | 7.57 | 0.94 | 0.17 | 2.62 | 0.94 | 0.17 | 2.62 | 0.99 | 0.03 | 4.33 | 1.00 | 0.00 | 0.00 | 0.99 | 0.03 | 2.58 | 0.98 | 0.17 | 6.99 | 0.93 | 0.17 | 2.48 | 0.93 | 0.19 | 2.68 |
|  |  | 0.5Mbp | 0.99 | 0.26 | 19.07 | 0.96 | 0.29 | 6.87 | 0.96 | 0.29 | 6.85 | 0.99 | 0.03 | 4.13 | 1.00 | 0.00 | 0.00 | 0.99 | 0.03 | 2.58 | 0.98 | 0.29 | 14.54 | 0.95 | 0.29 | 6.28 | 0.95 | 0.32 | 6.07 |
|  |  | 0.1Mbp | 0.97 | 0.26 | 9.51 | 0.90 | 0.39 | 3.69 | 0.90 | 0.39 | 3.69 | 0.99 | 0.03 | 4.78 | 0.99 | 0.00 | 0.00 | 0.98 | 0.03 | 2.10 | 0.97 | 0.29 | 9.05 | 0.89 | 0.39 | 3.42 | 0.88 | 0.42 | 3.57 |
|  |  | Adjacent | 0.97 | 0.33 | 12.00 | 0.90 | 0.37 | 3.51 | 0.90 | 0.37 | 3.51 | 0.99 | 0.03 | 4.68 | 0.99 | 0.00 | 0.00 | 0.99 | 0.03 | 2.74 | 0.97 | 0.37 | 10.92 | 0.89 | 0.37 | 3.36 | 0.89 | 0.40 | 3.50 |
|  |  | Nearest | 0.93 | 0.43 | 6.47 | 0.82 | 0.48 | 2.62 | 0.82 | 0.48 | 2.62 | 0.99 | 0.05 | 4.83 | 0.98 | 0.00 | 0.00 | 0.97 | 0.05 | 1.74 | 0.93 | 0.48 | 6.32 | 0.81 | 0.48 | 2.45 | 0.80 | 0.52 | 2.61 |
|  |  | Resident | 0.95 | 0.50 | 9.43 | 0.85 | 0.50 | 3.25 | 0.85 | 0.50 | 3.25 | 1.00 | 0.00 | 0.00 | 0.93 | 0.07 | 1.03 | 0.93 | 0.07 | 1.00 | 0.95 | 0.50 | 8.71 | 0.79 | 0.50 | 2.33 | 0.79 | 0.50 | 2.31 |
| MWS | K | 1Mbp | 0.98 | 0.33 | 15.34 | 0.91 | 0.42 | 4.67 | 0.91 | 0.42 | 4.67 | 0.99 | 0.00 | 0.00 | 0.94 | 0.00 | 0.00 | 0.93 | 0.00 | 0.00 | 0.97 | 0.33 | 10.87 | 0.86 | 0.42 | 2.88 | 0.85 | 0.42 | 2.71 |
|  |  | 0.5Mbp | 0.96 | 0.36 | 9.54 | 0.90 | 0.45 | 4.56 | 0.90 | 0.45 | 4.56 | 0.99 | 0.00 | 0.00 | 0.90 | 0.00 | 0.00 | 0.89 | 0.00 | 0.00 | 0.95 | 0.36 | 7.35 | 0.81 | 0.45 | 2.33 | 0.79 | 0.45 | 2.20 |
|  |  | 0.1Mbp | 0.94 | 0.50 | 7.49 | 0.89 | 0.67 | 5.62 | 0.88 | 0.67 | 5.38 | 0.99 | 0.00 | 0.00 | 0.76 | 0.00 | 0.00 | 0.75 | 0.00 | 0.00 | 0.94 | 0.50 | 7.49 | 0.65 | 0.67 | 1.89 | 0.64 | 0.67 | 1.86 |
|  |  | Adjacent | 0.92 | 0.50 | 6.03 | 0.85 | 0.60 | 3.89 | 0.85 | 0.60 | 3.89 | 0.99 | 0.00 | 0.00 | 0.88 | 0.00 | 0.00 | 0.87 | 0.00 | 0.00 | 0.92 | 0.50 | 5.69 | 0.73 | 0.60 | 2.19 | 0.72 | 0.60 | 2.15 |
|  |  | Nearest | 0.88 | 0.75 | 5.67 | 0.74 | 1.00 | 3.64 | 0.74 | 1.00 | 3.64 | 0.99 | 0.00 | 0.00 | 0.97 | 0.00 | 0.00 | 0.96 | 0.00 | 0.00 | 0.87 | 0.75 | 5.28 | 0.71 | 1.00 | 3.29 | 0.71 | 1.00 | 3.29 |
|  |  | Resident | 0.82 | 1.00 | 4.73 | 0.74 | 1.00 | 3.47 | 0.74 | 1.00 | 3.47 | 1.00 | 0.00 | 0.00 | 0.84 | 0.00 | 0.00 | 0.84 | 0.00 | 0.00 | 0.82 | 1.00 | 4.73 | 0.58 | 1.00 | 2.26 | 0.58 | 1.00 | 2.26 |
|  | W | 1Mbp | 0.98 | 0.24 | 13.90 | 0.92 | 0.34 | 4.55 | 0.92 | 0.34 | 4.55 | 0.99 | 0.03 | 4.04 | 0.96 | 0.00 | 0.00 | 0.95 | 0.03 | 0.71 | 0.98 | 0.28 | 11.31 | 0.88 | 0.34 | 2.99 | 0.88 | 0.38 | 3.10 |
|  |  | 0.5Mbp | 0.97 | 0.26 | 8.39 | 0.90 | 0.41 | 4.17 | 0.90 | 0.41 | 4.17 | 0.99 | 0.04 | 3.19 | 0.92 | 0.00 | 0.00 | 0.91 | 0.04 | 0.39 | 0.96 | 0.30 | 7.37 | 0.83 | 0.41 | 2.32 | 0.82 | 0.44 | 2.41 |
|  |  | 0.1Mbp | 0.94 | 0.35 | 6.07 | 0.88 | 0.55 | 4.49 | 0.88 | 0.55 | 4.39 | 0.99 | 0.00 | 0.00 | 0.78 | 0.00 | 0.00 | 0.77 | 0.00 | 0.00 | 0.94 | 0.35 | 6.07 | 0.66 | 0.55 | 1.63 | 0.66 | 0.55 | 1.61 |
|  |  | Adjacent | 0.92 | 0.43 | 4.98 | 0.84 | 0.57 | 3.55 | 0.84 | 0.57 | 3.55 | 0.99 | 0.05 | 5.62 | 0.88 | 0.00 | 0.00 | 0.88 | 0.05 | 0.38 | 0.91 | 0.48 | 5.37 | 0.73 | 0.57 | 2.08 | 0.72 | 0.62 | 2.23 |
|  |  | Nearest | 0.87 | 0.55 | 4.16 | 0.75 | 0.64 | 2.47 | 0.74 | 0.64 | 2.45 | 0.99 | 0.00 | 0.00 | 0.97 | 0.00 | 0.00 | 0.96 | 0.00 | 0.00 | 0.86 | 0.55 | 3.88 | 0.72 | 0.64 | 2.25 | 0.72 | 0.64 | 2.23 |
|  |  | Resident | 0.82 | 0.71 | 3.59 | 0.72 | 0.57 | 1.96 | 0.72 | 0.71 | 2.41 | 1.00 | 0.00 | 0.00 | 0.85 | 0.14 | 0.95 | 0.85 | 0.14 | 0.95 | 0.82 | 0.71 | 3.59 | 0.58 | 0.71 | 1.68 | 0.58 | 0.71 | 1.68 |
| MHS | K | 1Mbp | 0.97 | 0.43 | 11.68 | 0.90 | 0.43 | 4.20 | 0.90 | 0.43 | 4.20 | 0.99 | 0.00 | 0.00 | 0.78 | 0.00 | 0.00 | 0.77 | 0.00 | 0.00 | 0.96 | 0.43 | 9.60 | 0.68 | 0.43 | 1.36 | 0.68 | 0.43 | 1.32 |
|  |  | 0.5Mbp | 0.96 | 0.43 | 9.06 | 0.85 | 0.43 | 2.81 | 0.85 | 0.43 | 2.81 | 0.99 | 0.00 | 0.00 | 0.74 | 0.00 | 0.00 | 0.73 | 0.00 | 0.00 | 0.95 | 0.43 | 8.02 | 0.59 | 0.43 | 1.04 | 0.58 | 0.43 | 1.02 |
|  |  | 0.1Mbp | 0.93 | 0.75 | 8.85 | 0.89 | 0.75 | 6.32 | 0.89 | 0.75 | 6.32 | 1.00 | 0.00 | 0.00 | 0.59 | 0.00 | 0.00 | 0.59 | 0.00 | 0.00 | 0.93 | 0.75 | 8.85 | 0.48 | 0.75 | 1.43 | 0.48 | 0.75 | 1.43 |
|  |  | Adjacent | 0.90 | 0.40 | 3.87 | 0.88 | 0.40 | 3.10 | 0.87 | 0.40 | 2.93 | 0.99 | 0.00 | 0.00 | 0.71 | 0.00 | 0.00 | 0.70 | 0.00 | 0.00 | 0.90 | 0.40 | 3.87 | 0.58 | 0.40 | 0.96 | 0.58 | 0.40 | 0.94 |
|  |  | Nearest | 0.79 | 1.00 | 3.88 | 0.72 | 1.00 | 3.10 | 0.72 | 1.00 | 3.10 | 1.00 | 0.00 | 0.00 | 0.79 | 0.00 | 0.00 | 0.79 | 0.00 | 0.00 | 0.79 | 1.00 | 3.88 | 0.52 | 1.00 | 1.94 | 0.52 | 1.00 | 1.94 |
|  |  | Resident | 0.75 | 1.00 | 2.50 | 0.75 | 1.00 | 2.50 | 0.75 | 1.00 | 2.50 | 1.00 | 0.00 | 0.00 | 1.00 | 0.00 | 0.00 | 1.00 | 0.00 | 0.00 | 0.75 | 1.00 | 2.50 | 0.75 | 1.00 | 2.50 | 0.75 | 1.00 | 2.50 |
|  | W | 1Mbp | 0.97 | 0.30 | 10.19 | 0.90 | 0.45 | 4.46 | 0.90 | 0.45 | 4.46 | 0.99 | 0.05 | 8.12 | 0.81 | 0.00 | 0.00 | 0.81 | 0.05 | 0.26 | 0.97 | 0.35 | 9.83 | 0.72 | 0.45 | 1.59 | 0.71 | 0.50 | 1.73 |
|  |  | 0.5Mbp | 0.96 | 0.39 | 9.08 | 0.85 | 0.50 | 3.33 | 0.85 | 0.50 | 3.33 | 1.00 | 0.00 | 0.00 | 0.75 | 0.00 | 0.00 | 0.75 | 0.00 | 0.00 | 0.96 | 0.39 | 8.39 | 0.60 | 0.50 | 1.25 | 0.60 | 0.50 | 1.24 |
|  |  | 0.1Mbp | 0.94 | 0.50 | 7.39 | 0.90 | 0.50 | 4.88 | 0.90 | 0.50 | 4.88 | 1.00 | 0.00 | 0.00 | 0.57 | 0.00 | 0.00 | 0.57 | 0.00 | 0.00 | 0.94 | 0.50 | 7.39 | 0.47 | 0.50 | 0.95 | 0.47 | 0.50 | 0.95 |
|  |  | Adjacent | 0.92 | 0.58 | 6.78 | 0.88 | 0.50 | 4.15 | 0.88 | 0.58 | 4.68 | 1.00 | 0.08 | 19.38 | 0.72 | 0.00 | 0.00 | 0.71 | 0.08 | 0.29 | 0.92 | 0.58 | 6.78 | 0.60 | 0.50 | 1.25 | 0.60 | 0.58 | 1.44 |
|  |  | Nearest | 0.82 | 0.75 | 3.98 | 0.75 | 0.75 | 2.92 | 0.75 | 0.75 | 2.92 | 1.00 | 0.00 | 0.00 | 0.77 | 0.00 | 0.00 | 0.77 | 0.00 | 0.00 | 0.82 | 0.75 | 3.98 | 0.52 | 0.75 | 1.54 | 0.52 | 0.75 | 1.54 |
|  |  | Resident | 0.68 | 1.00 | 2.70 | 0.68 | 1.00 | 2.70 | 0.68 | 1.00 | 2.70 | 1.00 | 0.00 | 0.00 | 0.92 | 0.00 | 0.00 | 0.92 | 0.00 | 0.00 | 0.68 | 1.00 | 2.70 | 0.60 | 1.00 | 2.25 | 0.60 | 1.00 | 2.25 |
| HS | K | 1Mbp | 0.99 | 0.20 | 12.00 | 0.84 | 0.00 | 0.00 | 0.82 | 0.20 | 1.12 | 0.99 | 0.00 | 0.00 | 0.77 | 0.00 | 0.00 | 0.76 | 0.00 | 0.00 | 0.97 | 0.20 | 6.86 | 0.60 | 0.00 | 0.00 | 0.58 | 0.20 | 0.48 |
|  |  | 0.5Mbp | 0.95 | 0.25 | 5.19 | 0.88 | 0.00 | 0.00 | 0.86 | 0.25 | 1.73 | 0.99 | 0.00 | 0.00 | 0.66 | 0.25 | 0.73 | 0.64 | 0.25 | 0.70 | 0.94 | 0.25 | 4.15 | 0.53 | 0.25 | 0.54 | 0.50 | 0.50 | 1.00 |
|  |  | 0.1Mbp | 0.87 | 1.00 | 6.64 | 0.93 | 0.50 | 6.08 | 0.85 | 1.00 | 5.62 | 1.00 | 0.00 | 0.00 | 0.68 | 0.00 | 0.00 | 0.68 | 0.00 | 0.00 | 0.87 | 1.00 | 6.64 | 0.61 | 0.50 | 1.26 | 0.52 | 1.00 | 2.03 |
|  |  | Adjacent | 0.75 | 1.00 | 3.56 | 0.91 | 0.50 | 4.75 | 0.75 | 1.00 | 3.56 | 1.00 | 0.00 | 0.00 | 0.75 | 0.00 | 0.00 | 0.75 | 0.00 | 0.00 | 0.75 | 1.00 | 3.56 | 0.65 | 0.50 | 1.43 | 0.49 | 1.00 | 1.90 |
|  |  | Nearest | 0.00 | 1.00 | 1.00 | 0.00 | 1.00 | 1.00 | 0.00 | 1.00 | 1.00 | 1.00 | 0.00 | 0.00 | 1.00 | 0.00 | 0.00 | 1.00 | 0.00 | 0.00 | 0.00 | 1.00 | 1.00 | 0.00 | 1.00 | 1.00 | 0.00 | 1.00 | 1.00 |
|  |  | Resident | 1.00 | 0.00 | 0.00 | 1.00 | 0.00 | 0.00 | 1.00 | 0.00 | 0.00 | 1.00 | 0.00 | 0.00 | 1.00 | 0.00 | 0.00 | 1.00 | 0.00 | 0.00 | 1.00 | 0.00 | 0.00 | 1.00 | 0.00 | 0.00 | 1.00 | 0.00 | 0.00 |
|  | W | 1Mbp | 1.00 | 0.09 | 20.20 | 0.83 | 0.18 | 1.06 | 0.82 | 0.27 | 1.54 | 1.00 | 0.00 | 0.00 | 0.75 | 0.00 | 0.00 | 0.75 | 0.00 | 0.00 | 0.99 | 0.09 | 11.55 | 0.58 | 0.18 | 0.43 | 0.57 | 0.27 | 0.64 |
|  |  | 0.5Mbp | 0.97 | 0.22 | 6.88 | 0.87 | 0.11 | 0.88 | 0.87 | 0.22 | 1.67 | 1.00 | 0.00 | 0.00 | 0.64 | 0.11 | 0.31 | 0.64 | 0.11 | 0.31 | 0.97 | 0.22 | 6.19 | 0.51 | 0.22 | 0.46 | 0.50 | 0.33 | 0.67 |
|  |  | 0.1Mbp | 0.93 | 0.50 | 6.78 | 0.93 | 0.17 | 2.35 | 0.90 | 0.50 | 4.84 | 1.00 | 0.00 | 0.00 | 0.65 | 0.00 | 0.00 | 0.65 | 0.00 | 0.00 | 0.93 | 0.50 | 6.78 | 0.59 | 0.17 | 0.41 | 0.56 | 0.50 | 1.13 |
|  |  | Adjacent | 0.87 | 1.00 | 7.10 | 0.92 | 0.33 | 3.88 | 0.85 | 1.00 | 6.47 | 1.00 | 0.00 | 0.00 | 0.71 | 0.00 | 0.00 | 0.71 | 0.00 | 0.00 | 0.87 | 1.00 | 7.10 | 0.63 | 0.33 | 0.89 | 0.56 | 1.00 | 2.26 |
|  |  | Nearest | 0.61 | 1.00 | 2.43 | 0.55 | 1.00 | 2.13 | 0.55 | 1.00 | 2.13 | 1.00 | 0.00 | 0.00 | 1.00 | 0.00 | 0.00 | 1.00 | 0.00 | 0.00 | 0.61 | 1.00 | 2.43 | 0.55 | 1.00 | 2.13 | 0.55 | 1.00 | 2.13 |
|  |  | Resident | 1.00 | 0.00 | 0.00 | 1.00 | 0.00 | 0.00 | 1.00 | 0.00 | 0.00 | 1.00 | 0.00 | 0.00 | 1.00 | 0.00 | 0.00 | 1.00 | 0.00 | 0.00 | 1.00 | 0.00 | 0.00 | 1.00 | 0.00 | 0.00 | 1.00 | 0.00 | 0.00 |

**Table S5 LD versus naïve clustering**

| **Disease** | **HS** |  |  | **MHS** |  |  |
| --- | --- | --- | --- | --- | --- | --- |
|  | **SNPs** | **LD loci** | **Clusters** | **SNPs** | **LD loci** | **Clusters** |
| Bipolar disorder (BD) | - | - | - | 23 | 7 | 6 |
| Coronary artery disease (CAD) | 22 | 10 | 10 | 38 | 21 | 21 |
| Crohn’s disease (CD) | 62 | 8 | 10 | 101 | 17 | 16 |
| Hypertension (HT) | - | - | - | 5 | 5 | 5 |
| Rheumatoid arthritis (RA) | 11 | 2 | 5 | 26 | 8 | 8 |
| Type I diabetes (T1D) | 91 | 9 | 24 | 160 | 15 | 15 |
| Type II diabetes (T2D) | 16 | 2 | 2 | 40 | 13 | 12 |

LD loci refers to the number of significant regions defined by linkage disequilibrium from the significantly associated SNPs (r2 > 0.5, CEU population). Clusters refers to the number of naïve SNP clusters formed by clustering significantly associated SNPs within 50Kbp of one another.

**Table S6 Comparison of the number of significant *Gentrepid* predictions between LD and adjacent mapping gene sets**

| **Disease** | **HS** |  |  |  | **MHS** |  |  |  |
| --- | --- | --- | --- | --- | --- | --- | --- | --- |
|  | **LD** |  | ***Adjacent*** |  | **LD** |  | ***Adjacent*** |  |
|  | **N** | **P** | **N** | **P** | **N** | **P** | **N** | **P** |
| Bipolar disorder (BD) | *-* | *-* | *-* | *-* | 15 | 2 | 28 | 0 |
| Coronary artery disease (CAD) | 19 | 0 | 36 | 2 | 39 | 5 | 71 | 3 |
| Crohn’s disease (CD) | 31 | 2 | 34 | 3 | 96 | 4 | 75 | 0 |
| Hypertension (HT) | - | - | - | - | 5 | 0 | 18 | 0 |
| Rheumatoid arthritis (RA) | 15 | 3 | 11 | 4 | 35 | 6 | 41 | 4 |
| Type I diabetes (T1D) | 218 | 24 | 96 | 16 | 234 | 38 | 157 | 41 |
| Type II diabetes (T2D) | 3 | 1 | 6 | 1 | 19 | 2 | 46 | 2 |

Each value in the table represents the number of genes. Abbreviations: N, size of search space; P, number of predictions.

**Table S7 Total number of significant predictions across *Gentrepid* , GRAIL and WebGestalt.**

| **Disease** | **HS** |  |  |  | **MHS** |  |  |  |
| --- | --- | --- | --- | --- | --- | --- | --- | --- |
|  | ***Gentrepid*** | **GRAIL** | **WebGestalt** | **LD search space size** | ***Gentrepid*** | **GRAIL** | **WebGestalt** | **LD search space size** |
| Bipolar disorder (BD) | - | - | - | - | 0 | 3 | 6 | 15 |
| Coronary artery disease (CAD) | 0 | 3 | 6 | 19 | 3 | 9 | 13 | 39 |
| Crohn’s disease (CD) | 0 | 5 | 18 | 31 | 3 | 26 | 48 | 96 |
| Hypertension (HT) | - | - | - | - | 0 | 1 | 5 | 5 |
| Rheumatoid arthritis (RA) | 2 | 9 | 7 | 15 | 6 | 15 | 19 | 35 |
| Type I diabetes (T1D) | 18 | 81 | 82 | 218 | 18 | 104 | 104 | 234 |
| Type II diabetes (T2D) | 1 | 2 | 1 | 3 | 1 | 7 | 4 | 19 |

Each value in table represents number of genes. There were no significant loci in BD and HT.

**Table S8 Specificity, Sensitivity and Enrichment ratios for WTCCC validation set for *Gentrepid*, GRAIL and WebGestalt**

|  |  | **MHS set** | | | | | | **HS set** | | | | | |
| --- | --- | --- | --- | --- | --- | --- | --- | --- | --- | --- | --- | --- | --- |
|  |  | *p < 0.05* | | | *p < 0.01* | | | *p < 0.05* | | | *p < 0.01* | | |
| **System** |  | **Sp** | **Se** | **ER** | **Sp** | **Se** | **ER** | **Sp** | **Se** | **ER** | **Sp** | **Se** | **ER** |
| ***Gentrepid*** |  |  |  |  |  |  |  |  |  |  |  |  |  |
|  | CMP-s* | 0.93 | 0.03 | 0.49 | 1.00 | 0.00 | 0.00 | 0.95 | 0.10 | 1.70 | 1.00 | 0.00 | 0.00 |
|  | CPS-s | 0.98 | 0.26 | 6.72 | 0.99 | 0.19 | 7.15 | 0.98 | 0.19 | 6.81 | 1.00 | 0.05 | 13.62 |
|  | CMP-ab* | 0.73 | 0.26 | 0.97 | 0.93 | 0.03 | 0.49 | 0.91 | 0.29 | 2.64 | 0.95 | 0.10 | 1.70 |
|  | CPS-ab | 0.95 | 0.26 | 3.81 | 0.97 | 0.26 | 5.44 | 0.94 | 0.10 | 1.51 | 0.96 | 0.05 | 1.13 |
| **GRAIL** |  |  |  |  |  |  |  |  |  |  |  |  |  |
|  | PUB-s | 0.98 | 0.06 | 2.86 | 1.00 | 0.06 | 14.29 | 1.00 | 0.00 | 0.00 | 1.00 | 0.00 | 0.00 |
|  | PUB2-s | 0.97 | 0.16 | 3.76 | 1.00 | 0.10 | 10.72 | 0.97 | 0.14 | 4.09 | 1.00 | 0.10 | 13.62 |
|  | GO-G-s | 0.99 | 0.00 | 0.00 | 1.00 | 0.00 | 0.00 | 0.99 | 0.00 | 0.00 | 1.00 | 0.00 | 0.00 |
|  | HEA-s | 0.95 | 0.00 | 0.00 | 1.00 | 0.00 | 0.00 | 0.94 | 0.00 | 0.00 | 0.99 | 0.00 | 0.00 |
|  | PUB | 0.86 | 0.42 | 2.69 | 0.93 | 0.29 | 3.48 | 1.00 | 0.00 | 0.00 | 1.00 | 0.00 | 0.00 |
|  | PUB2 | 0.82 | 0.45 | 2.30 | 0.89 | 0.32 | 2.60 | 0.78 | 0.29 | 1.30 | 0.84 | 0.19 | 1.16 |
|  | GO-G | 0.94 | 0.29 | 4.02 | 0.99 | 0.16 | 8.93 | 0.93 | 0.10 | 1.30 | 0.98 | 0.05 | 2.27 |
|  | HEA | 0.95 | 0.06 | 1.19 | 0.99 | 0.03 | 2.86 | 0.96 | 0.10 | 2.10 | 0.98 | 0.05 | 2.27 |
| **WebGestalt** |  |  |  |  |  |  |  |  |  |  |  |  |  |
|  | GO-W | 0.92 | 0.16 | 1.83 | 0.95 | 0.00 | 0.00 | 0.99 | 0.10 | 5.45 | 1.00 | 0.00 | 0.00 |
|  | KEGG | 0.89 | 0.23 | 1.89 | 0.93 | 0.23 | 2.86 | 0.90 | 0.29 | 2.48 | 0.92 | 0.14 | 1.70 |
|  | WIKI | 0.92 | 0.32 | 3.40 | 0.96 | 0.19 | 3.57 | 0.93 | 0.38 | 4.19 | 0.95 | 0.19 | 3.40 |
|  | PATHS | 0.94 | 0.26 | 3.69 | 0.99 | 0.16 | 7.15 | 0.95 | 0.24 | 4.01 | 0.99 | 0.05 | 4.54 |
|  | PPI | 0.91 | 0.16 | 1.74 | 0.92 | 0.13 | 1.63 | 0.88 | 0.10 | 0.83 | 0.91 | 0.10 | 1.01 |
|  | TF | 0.89 | 0.16 | 1.43 | 0.97 | 0.00 | 0.00 | 0.89 | 0.19 | 1.60 | 0.96 | 0.14 | 2.92 |
|  | MIR | 0.94 | 0.06 | 1.10 | 0.98 | 0.06 | 2.38 | 0.97 | 0.14 | 3.71 | 0.98 | 0.10 | 3.89 |

*Do not correspond with the significance thresholds specified. For CMP-s, instead of 0.05, we used a score of 0.4 and instead of 0.01, a score of 0.8. For CMP-ab, 100 and 10^5^.

Abbreviations: ab – ab initio, s – seeded, Sp – specificity, Se- sensitivity, ER – enrichment ratio. Refer to text for calculations.

CMP and CPS are *Gentrepid* methods. PUB, PUB2, GO-G, and HEA are GRAIL methods. PUB refers to PubMed abstracts up until 2006. PUB2 refers to PubMed abstracts up until 2011. GO-G refers to the gene ontology GRAIL method. HEA refers to the human expression atlas. GO-W, KEGG, WIKI, PATHS, PPI, TF and MIR refer to the methods of WebGestalt. GO-W refers to gene ontology enrichment. KEGG refers to pathway enrichment from the KEGG database. WIKI refers to pathway enrichment from Wikipathways. PATHS refers to pathway enrichment from Pathway Commons. PPI refers to protein interaction enrichment. TF refers to transcription factor enrichment. MIR refers to microRNA enrichment. CPS has the most consistent results.
