# Supplementary figures and images for: Analysis of genome-wide association study data using the protein knowledge base
Source: BMC Genet. 2011 Nov 13;12:98. doi: 10.1186/1471-2156-12-98 (PMC3261104; doi:10.1186/1471-2156-12-98)

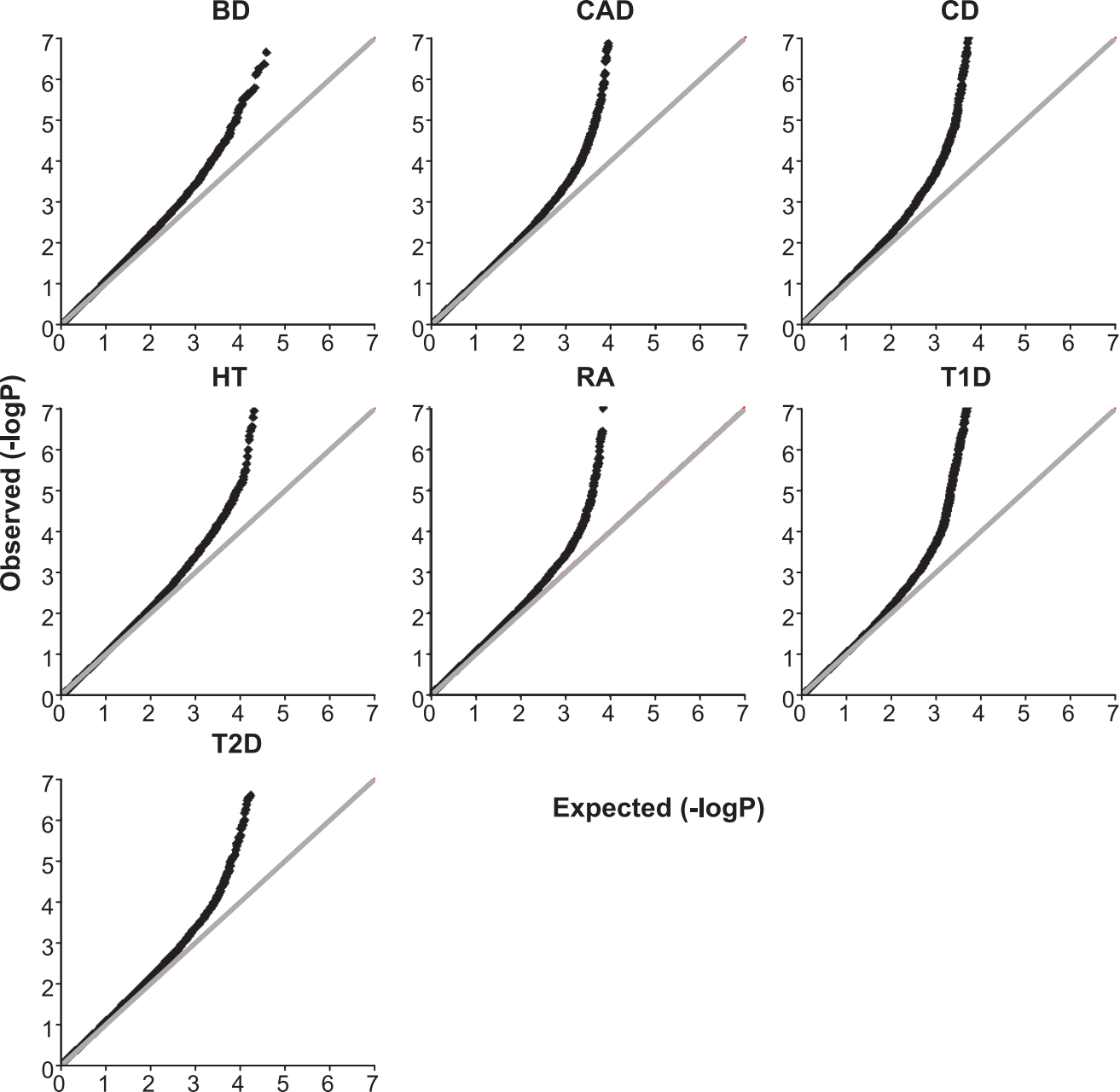

Supplement: Additional file 2 — Figure S1 Q-Q plots of expected values of the associated trend test p-values versus observed generated for each phenotype in black and uniform distribution in grey. [file 1471-2156-12-98-S2.pdf]

Known set

WTCCC set

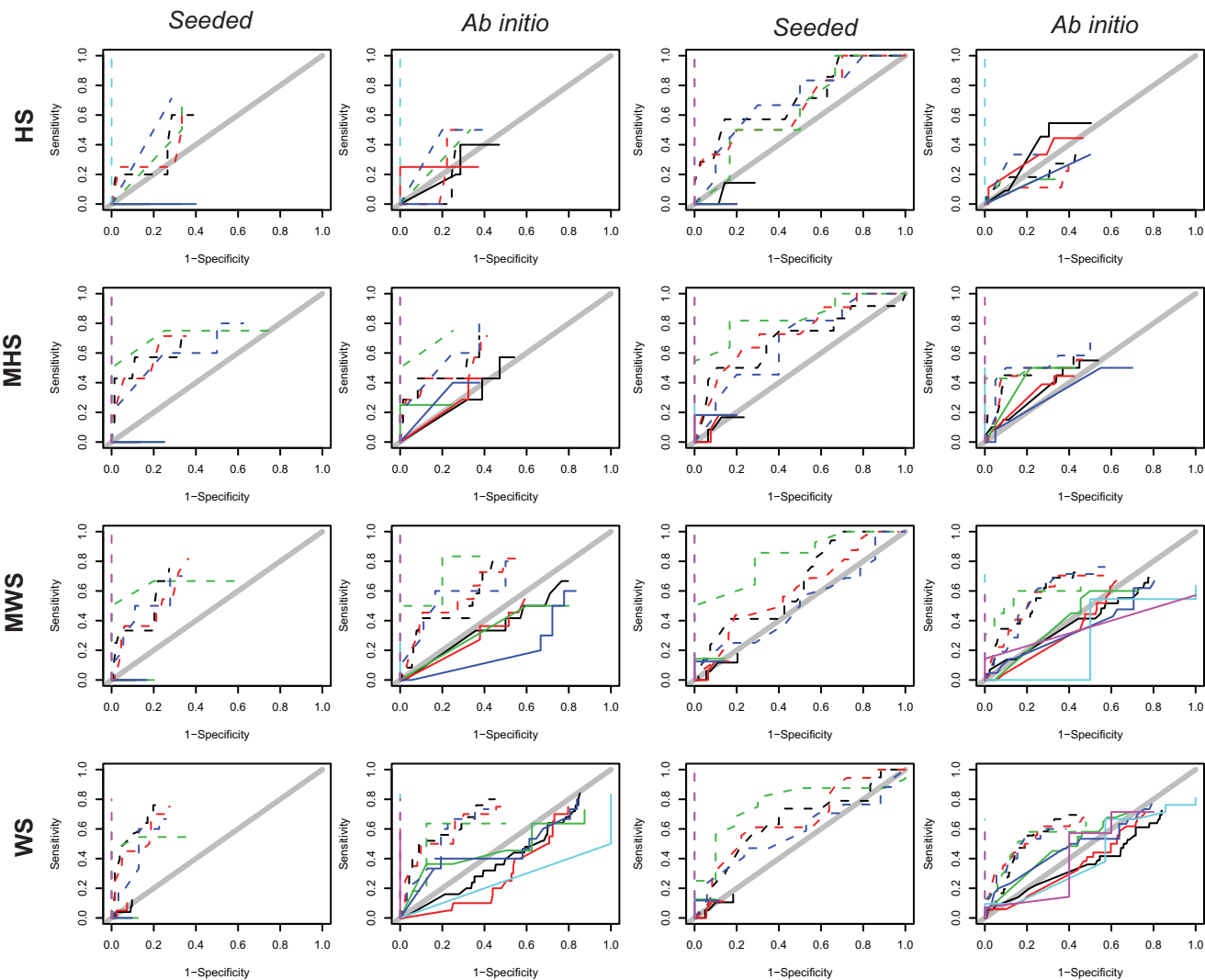

-- CPS  
— CMP  
— 1Mbp  
— 0.5Mbp  
— 0.1Mbp  
— Adjacent  
— Nearest  
— Resident

Supplement: Additional file 3 — Figure S2 ROC curves for Gentrepid on known and WTCCC validation sets. CPS is represented by the dashed lines, CMP by the filled lines. The colors indicate the SNP-to-gene mapping set used. The first column from the left are the results for the known validation set using seeded mode, The second column are the known validation set under ab initio. The third column is the WTCCC validation set seeded results. And the fourth column the WTCCC set, ab initio. The top panels are the HS sets. The next set of panels the MHS set, the third MWS and the bottom panels the WS set. The grey line in each plot represents what a random guess should give. CPS is above the line for most cases. CMP is below. CPS with the 0.1 Mbp or adjacent set performs the best. [file 1471-2156-12-98-S3.pdf]
